# Trematode *Diplostomum pseudospathaceum* inducing differential immune gene expression in sexual and gynogenetic gibel carp (*Carassius gibelio)*: parasites facilitating the coexistence of two reproductive forms of the invasive species

**DOI:** 10.3389/fimmu.2024.1392569

**Published:** 2024-06-25

**Authors:** Md Mehedi Hasan Fuad, Tomáš Tichopád, Markéta Ondračková, Kristína Civáňová Křížová, Mária Seifertová, Kristýna Voříšková, Martin Demko, Lukáš Vetešník, Andrea Šimková

**Affiliations:** ^1^ Department of Botany and Zoology, Faculty of Science, Masaryk University, Brno, Czechia; ^2^ Laboratory of Non-Mendelian Evolution, Institute of Animal Physiology and Genetics of the Czech Academy of Science, Liběchov, Czechia; ^3^ University of South Bohemia in České Budějovice, Faculty of Fisheries and Protection of Waters, South Bohemian Research Center of Aquaculture and Biodiversity of Hydrocenoses, Vodňany, Czechia; ^4^ Institute of Vertebrate Biology of the Czech Academy of Sciences, Brno, Czechia; ^5^ Central European Institute of Technology, Masaryk University, Brno, Czechia

**Keywords:** parasites, fish, RNA seq, differential gene expression, immunity-associated pathways, invasive species, asexual and sexual reproduction

## Abstract

**Introduction:**

Parasite-mediated selection is considered one of the potential mechanisms contributing to the coexistence of asexual-sexual complexes. Gibel carp (*Carassius gibelio*), an invasive fish species in Europe, often forms populations composed of gynogenetic and sexual specimens.

**Methods:**

The experimental infection was induced in gynogenetic and sexual gibel carp using eye-fluke *Diplostomum pseudospathaceum* (Trematoda), and the transcriptome profile of the spleen as a major immune organ in fish was analyzed to reveal the differentially expressed immunity-associated genes related to *D. pseudospathaceum* infection differing between gynogenetic and sexual gibel carp.

**Results:**

High parasite infection was found in gynogenetic fish when compared to genetically diverse sexuals. Although metacercariae of *D. pseudospathaceum* are situated in an immune-privileged organ, our results show that eye trematodes may induce a host immune response. We found differential gene expression induced by eye-fluke infection, with various impacts on gynogenetic and sexual hosts, documenting for the majority of DEGs upregulation in sexuals, and downregulation in asexuals. Differences in gene regulation between gynogenetic and sexual gibel carp were evidenced in many immunity-associated genes. GO analyses revealed the importance of genes assigned to the GO terms: immune function, the Notch signaling pathway, MAP kinase tyrosine/threonine/phosphatase activity, and chemokine receptor activity. KEGG analyses revealed the importance of the genes involved in 12 immunity-associated pathways – specifically, FoxO signaling, adipocytokine signaling, TGF-beta signaling, apoptosis, Notch signaling, C-type lectin receptor signaling, efferocytosis, intestinal immune network for IgA production, insulin signaling, virion - human immunodeficiency virus, Toll-like receptor signaling, and phosphatidylinositol signaling system.

**Discussion:**

Our study indicates the limited potential of asexual fish to cope with higher parasite infection (likely a loss of capacity to induce an effective immune response) and highlights the important role of molecular mechanisms associated with immunity for the coexistence of gynogenetic and sexual gibel carp, potentially contributing to its invasiveness.

## 1 Introduction

Gibel carp (*Carassius gibelio* (Bloch, 1782)) is a widely-distributed invasive cyprinid species in European freshwaters (1, 2). It is one of the few vertebrates among fish, amphibians and lizards that exhibit a diverse ploidy level and a capacity for both the sexual and asexual modes of reproduction (3, 4). Although until the 1990s, European populations of gibel carp were presumed to comprise triploid females (5, 6) which reproduced asexually, both sexual and asexual forms of gibel carp have, since then, been reported in natural habitats (5). A unique ability to reproduce rapidly through the gynogenesis as a specific mode of asexual reproduction places gibel carp in the role of a successful invasive species (7). In gynogenesis, the eggs are activated by the sperm of conspecific males or closely-related species (often common carp); however, due to the absence of syngamy, there is no genetic contribution of males to the progeny (8).

The mechanisms promoting the co-existence of asexual and sexual fish have been investigated mostly in *Phoxinus* (9, 10), *Poecilia* (11–14), *Cobitis* (15, 16), *Carassius auratus* (17, 18), and *C. gibelio* (8, 19, 20). Several mechanisms that facilitate the coexistence of sexual and gynogenetic reproductive forms of gibel carp have already been investigated and proposed. Gynogenetic triploid females exhibited a higher growth rate and better body condition expressed by total proteins in blood than sexual diploids (21, 22). However, Šimková et al. (20) showed lower aerobic performance in gynogenetic females compared to sexual specimens and suggested that this physiological disadvantage on the part of gynogens that may balance the costs of sexual reproduction. Vetešník et al. (22) revealed other disadvantages faced by gynogenetic females such as higher concentrations of triacylglycerols and cholesterol, which may indicate a higher metabolic rate, and higher energy intake when compared to sexual diploids. Interestingly, both gynogenetic and sexual females invested a similar amount of energy in reproduction, measured by the relative size of gonads and estradiol level (20). Although Šimková et al. (20) suggested that both gynogenetic and sexual females of gibel carp invested a similar amount of energy in reproduction, Xie et al. (23) showed differences in gene expression between oocytes of gynogenetic females and those of sexual females, suggesting the different behavior of eggs toward sperm.

Parasite-mediated selection was also suggested to contribute to the coexistence of sexual and gynogenetic gibel carp (19, 24). Differences in terms of immune response and susceptibility to parasitic infections were found between the gynogenetic and sexual forms. A higher specific immunity response (measured by IgM level) was found in gynogenetic females in contrast to their sexual counterparts (20). A study on the variability of major histocompatibility complex (MHC) genes in parasite-infected gynogenetic and sexual gibel carp revealed that the most common MHC genotypes of the gynogenetic females suffered from high parasite abundance or high metazoan species richness (mostly referring to host-specific monogeneans of the genus *Dactylogyrus*) (19). This is in line with the Red Queen hypothesis predicting that coevolving hosts and parasites are under negative frequency-dependent selection, and, thus, that asexual hosts (especially those originating from the clonal reproduction of one female specimen) are an ideal target for parasite adaptation, whilst sexual hosts due to genetic variability may escape parasitism (3, 25–28). A four-year study of parasite infection in gibel carp suggested that the Red Queen hypothesis is not the mechanism responsible for metazoan parasite infection in gynogenetic and sexual gibel carp over the investigated temporal scale (24); however, the need for immunological genotyping to verify this conclusion was highlighted. It was also shown that even the non-specific immune response is more effective in gynogenetic females when compared to sexual ones in the Japanese crucian carp (*C. auratus*), and, at the same time, that the prevalence of *Metagonimus* sp. (Trematoda), for which fish act as the second intermediate host, was higher in gynogenetic females than in sexual forms (17). Using theoretical models, Hakoyama and Iwasa (18) suggested that parasitism plays a role in realizing the coexistence of asexual-sexual complexes by providing a frequency-dependent benefit to sexual populations; however, they emphasized that parasite virulence is an important parameter in a coexistence-predicting model.

*Diplostomum pseudospathaceum* Niewiadomska 1984 is a widely-distributed digenean trematode parasitizing the eye lens of the fish (29). Based on the Host-parasite database of Natural of Natural History Museum, London, (https://www.nhm.ac.uk/research-curation/scientific-resources/taxonomy-systematics/host-parasites/), this parasite was reported from 20 fish host species. The complex life cycle of this trematode includes two intermediate hosts (aquatic snails as the first intermediate host and fresh- or brackish-water fish as the second intermediate host) and piscivorous birds as definitive host (30). The adult parasites reproduce sexually in the intestine of piscivorous birds and release eggs. In the aquatic environment, free-swimming miracidia hatch from the eggs and infect the first intermediate host, aquatic snails. Within the snail body, thousands of cercariae are produced through asexual reproduction and released into the water. Cercariae infect fish by skin penetration, migrate to the eye lens, and transform to metacercariae. Metacercariae of *Diplostomum* eye flukes may damage the eye retina, which may lead to cataract of the lens (31). Apart from eye damage, a number of red patches and swelling can be observed on fins, and on body or eye areas in cases of heavy infection. In some cases, a high number of diplostomulas can block the blood vessels of gills causing asphyxia, shock, and damage to the nervous system (32).

Pathological effects in fish heavily parasitized by lens-infecting *Diplostomum* metacercariae include stunted growth, abnormal feeding due to the loss of visual acuity, and abnormal vitals (33), as well as increased mortality (34). Parasites modify fish host behavior by inducing surface seeking (35, 36), reducing escape response (36, 37), or altering shoaling (36, 38), and competitive (39) and reproductive (40) behavior. The infection either reduces or increases the aggressiveness of the fish, which ultimately makes them less vigilant and more conspicuous to visual predators (36, 41, 42).

As this eye-fluke inhabits an immune-privileged area – specifically, the eye, which is considered to be free of inflammatory cells and lymphocytes – the parasites have a better chance of survival (43). Furthermore, due to the parasite’s localization in the eye, the immune response is limited to the migratory period between epidermal penetration by the cercariae and their arrival in the eye. It is thus generally accepted that the classical adaptive response plays no role in resistance to a primary parasite infection (44) and that only the innate immunity of fish is activated against *D. pseudospathaceum* (29). However, both the innate and adaptive immunity of fish against this parasite have been reported. Macrophage activity has been described as the first response to *D. pseudospathaceum* infection (45), with macrophages producing reactive oxygen species capable of killing *Diplostomum* larvae (46). The importance of complement-fixing antibodies and cell-mediated immunity jointly or exclusively in adaptive immunity against *Diplostomum spathaceum* was described by Woo (47). A more detailed study on the transcriptomic response to *Diplostomum* infection in the three-spined stickleback showed the upregulation of five immune genes, suggesting an important role for enhanced Toll-like receptor activity (*ctsk*, *cyp27b1*) and an associated positive regulation of macrophages (*cyp27b1*, *thbs1*) in defense against eye-fluke infection (48). Additionally, many studies have shown that fish that survived *Diplostomum* infection or underwent experimental injection by sonicated parasites are protected from disease if re-exposed to the parasite in the future (e.g (29, 46, 47, 49)).

In the present study, gynogenetic and sexual specimens of *C. gibelio* were experimentally infected with cercariae of the eye-fluke trematode *D. pseudospathaceum* (infection by mixed genotypes). Based on the hypothesis that parasite-mediated selection plays an important role in the coexistence of sexual and asexual forms in nature, the present study focused on transcriptome profile analysis of the spleen (one of the major immune organs in fish) of gynogenetic and sexual gibel carp to reveal the differentially expressed immune genes related to infection with *D. pseudospathaceum*.

## 2 Material and methods

### 2.1 Fish selection and establishing parasitic infection

A total of 12 sexual and 12 gynogenetic immunologically naïve specimens of *Carassius gibelio* obtained by artificial breeding were selected for experimental infection by *Diplostomum pseudospathaceum*, and six sexual and six gynogenetic non-infected fish were used as a control. Asexual females were obtained by induced embryogenesis using sperm of common carp (*Cyprinus carpio*). Sexual specimens were obtained from the interbreeding of sexual specimens. The fish were reared in aquarium conditions until the age of one year (body weight 3.25 ± 0.46 g) and then used for experimental infection. Fish were fed with frozen adult artemia, the commercial dry pellet (www.Exothobby.cz), and flakes (Tetra Min) during the whole experiment.

Specimens of *Lymnaea stagnalis*, the first intermediate hosts of *D. pseudospathaceum*, were collected from the Vlkovsky pond, located near the town of Veselí nad Lužnicí in the Czech Republic (49.147679, 14.731210). Snail individuals infected with *Diplostomum* cercariae were separated and individually marked. Released cercariae were preserved in 96% ethanol and identified to species level using molecular methods involving the DNA sequencing of the COI and ITS1–5.8S-ITS2 regions (50). Prior to experimental infection, ten *L. stagnalis* infected with *D. pseudospathaceum* were allowed to release cercariae for two hours. Subsequently, the experimental fish were individually exposed to an infection dose of 200 cercariae for two hours at 20.5 - 21°C in 200 ml of dechlorinated water. Specifically, each fish specimen was exposed to the infection of the mixed genotypes of *D. pseudospathaceum*, i.e., cercariae from different snails were randomly mixed to ensure the diversity of different genotypes, and then 200 cercariae were randomly selected from the mix and used for infection of each individual fish. We assume that all genotypes were present in each infection dose per fish.

After exposure, fish were transported back to the aquaria with a comparable water temperature and kept for six weeks. Three days post infection, two fish per group were dissected to control for infection success. Six weeks post infection, after metacercariae had developed sufficiently to be infective for the definitive host (38), the next 20 experimental fish, including ten sexual and ten gynogenetic specimens, were dissected. The fish were killed by severing the cervical spine and the spleen was aseptically isolated and preserved in RNAlater solution (Sigma-Aldrich). The eyes were removed, and parasites were counted using an Olympus SZX 10 stereomicroscope. Parasite abundance and intensity of infection were calculated following Bush et al. (51). Simultaneously with the dissection of infected fish, we individually preserved the spleen of six sexual and six gynogenetic immunologically naïve specimens as a control.

### 2.2 RNA isolation, cDNA libraries and NGS sequencing

The spleens of individual fish were used for transcriptome profile analysis and subsequent differential gene expression analysis. Total RNA was isolated from each individually preserved spleen using PureLink^®^ RNA Mini Kit (Ambion) along with TRIzol™ Reagent (Invitrogen) and PureLink^®^ DNase (Ambion). The quantity of extracted RNA was assessed using a NanoDrop 8000 spectrophotometer (Thermo Fisher Scientific). The quality and integrity of RNA were analyzed using RNA 6000 Nano Kit on a 2100 Bioanalyzer instrument (Agilent Technologies).

RNA samples with an acceptable RNA integrity number (RIN > 7) were used for DNA library preparation. 500 ng of total RNA was used for mRNA enrichment using the Poly(A) mRNA Magnetic Isolation Module (New England Biolabs). Subsequently, NEBNext Ultra II Directional RNA Library Prep Kit for Illumina, and Dual Index Primers Set 2 of NEBNext Multiplex Oligos for Illumina (New England Biolabs) were used for library preparation. The numbers of PCR cyclers used for enrichment and the beads ratio used for fragment size selection were modified according to the recommendations in the manufacturers´ protocol. The quantification of DNA libraries was performed on a Qubit 4 fluorometer (Invitrogen by Thermo Fisher Scientific) using Qubit dsDNA HS Assay Kit, and quality and size controls were performed on a 2100 Bioanalyzer with DNA 1000 Kit (Agilent Technologies). Finally, amplicons (average size of 355 bp) were pooled in equimolar amounts. The final concentration of each library was 10 nM in the pool. Subsequently, the prepared cDNA libraries of 18 specimens, including 5 infected sexual, 5 infected gynogenetic, 4 sexual control and 4 gynogenetic control were sequenced by Macrogen Inc. (Republic of Korea) on an Illumina HiSeq X (one lane) in a paired-end configuration producing 150 bp long reads. Quality and quantity control steps were carried out by this service company.

### 2.3 NGS data pre-processing, differential gene expression, and GO term analyses

The quality control of raw paired-end fastq reads was initially performed using FastQC v0.12.1 (52). A simple trimming process was executed using BBTools (bbduk.sh) from the BBMap package version 39.06 (53). The trimming parameters were set to remove adapters and low-quality sequences. Specifically, the parameters included ftm=5, ktrim=r, k=23, mink=11, hdist=1, tpe, tbo, qtrim=rl, trimq=10, and minlen=40. The adapter sequences used for trimming were obtained from the BBTools resources (adapters.fa file).

After trimming, a second round of filtering was conducted to remove rRNA contamination. This step utilized BBTools with k=21 and a reference file containing ribosomal RNA sequences (ribokmers.fa.gz). Quality control checks on the processed reads were again performed using FastQC version v0.12.1 (52). RNAseq data, i.e., numbers of input reads, uniquely- and multi-mapped reads per sample are shown in **Supplementary Table 1**.

For the alignment process, STAR aligner version 2.7.11a (54) was used. Prior to read mapping, a STAR genome index was created using the *C. auratus* genome (Carassius_auratus.ASM336829v1.dna.toplevel.fa) and its corresponding GTF file (Carassius_auratus.ASM336829v1.110.chr.gtf) from the ENSEMBL depository. The STAR alignment was performed using the default settings, including the –sjdbOverhang 149 and –outSAMstrandField intronMotif options, achieving approximately 97% of reads mapped to the genome and about 89% mapping uniquely. After alignment, the featureCounts tool from the SUBREAD package version 2.0.6 (55) was used to quantify gene expression. This step involved counting read pairs (–p, –countReadPairs) and specifying the strand-specificity (-s 2). The gene annotations were provided through the *C. auratus* GTF file, and the counting was performed across all aligned BAM files.

For differential gene expression analysis, we used gene counts from the featureCounts tool and differentially expressed genes (DEGs) were determined by DESeq2 version 1.42.0 (56) in R 4.3.2 (57). An interaction term for reproduction (gynogenetic vs. sexual) and treatment (infected vs. control) was included in the model to investigate their combined impact on gene expression. The significance of DEGs between the experimental groups was calculated with an adjusted *P* value ≤ 0.05 (58) together with log_2_(fold change) values > 1 as a threshold for significance. For comparative purpose, we also analyzed DEGs including only the effect of treatment (infected vs. control) to see the immunity-associated genes having the same impact in gynogenetic and sexual fish. To analyze the biological significance of genes affected by the interaction between ploidy and treatment, we conducted Gene Set Enrichment Analysis (GSEA) using the ‘fgsea’ R package v 1.28.0 (59). Gene sets were ranked on the basis of the product of log_10_ (p-value) and the sign of their log_2_(fold change) values. This approach enabled the identification of key biological pathways impacted by the interaction, offering insights into the underlying cellular mechanisms. The GO enrichment was visualized using Revigo (60). We used p-values for semantic similarity in default settings (corresponding to medium mode (0.7)) against *Danio rerio* UniProt database.

For the KEGG Pathway enrichment analysis of DEGs in the goldfish (*C. auratus*) genome, DEGs were first converted to Entrez IDs using the biomaRt R package (version 2.58.0) (61). These IDs were then analyzed via the clusterProfiler R package (version 4.10.0) (62) and the function enrichKEGG in default setting, targeting the Goldfish KEGG database (the organism code ‘caua’). The analysis incorporated the Benjamini-Hochberg method for adjusting p-values, with a cutoff set at 0.05, and normalized enrichment score, |NES| > 1, to identify significant biological pathways and processes related to the DEGs.

### 2.4 Experimental corroboration of DEGs using quantitative real time PCR

RNA isolates of samples were normalized by dilution at a uniform concentration of 10 ng/µl with RNase-free water. They served as templates for the reverse transcription of total RNA into single-stranded cDNA using High-Capacity RNA-to-cDNA Kit (Applied Biosystems by Thermo Fisher Scientific) in twice the reaction volume recommended by the manufacturer. Prepared cDNA was diluted uniformly in all tested samples and served as a template for quantitative PCR (qPCR) analysis.

Fifteen immunity-related genes were selected from the list of DEGs based on RNA-seq but only eleven of them were successfully quantified using qPCR. The list of the selected genes is included in **Table 1**. The sequences of the selected DEGs were retrieved from the transcriptomic data, and then primers were designed using the primer designing tool of NCBI (https://www.ncbi.nlm.nih.gov/tools/primer-blast). The primers were designed to span an exon-exon junction.

**Table 1:** Immune gene selected for qPCR validation.

Gene name	Ensembl ID (ENSCARG)	Forward primer sequence (5´- 3´)	Reverse primer sequence (5´- 3´)	Amplicon size (bp)	Annealing temperature (°C)
*ccr9a*	00000001258	CCCCGTTCACTGGGATAGC	AACCGTCTTCATAATCACCCA	75	60
*cxcr4b*	00000006820	CGGGAGATCCGTTGACTGAA	GCTGTTGTCTAAAATGATGTGATCG	106	non amplified
*ddit4*	00000007230	ACATTAACGTGCATAATATCGTCAT	CGATGTTGGTGTGGATGGGA	105	55
*il6*	00000005779	GCGAGACCAGCAGTTTAAGAG	CTGTAAATGCGGCCCAGACA	150	50
*irf2bp2a*	00000029246	GCGTCCAAATCAGATCGAGG	TTTTCAATCCGTCTCCCTGC	139	non amplified
*itgb3a*	00000020529	GATAGAAGTTTGGGCGACAGTT	CCTCCCTTTCCAAAGATCTCC	150	55
*map3k8*	00000012648	TTTGGGAAGGTCCATCTGGC	GCTTGGATTTCCACGTCTGC	104	55
*nfkbiab*	00000004807	CGAGGCCACAGAGGCTG	CCAAATGCAATGCAGTCTGTC	98	55
*notch1b*	00000036847	GTTTACTTTGACGCATGGCCT	TTACGGAACTGGCACGCTG	132	non amplified
*pik3r1*	00000022851	CCAGCGAATGGAGTGACAGT	TTGACCTCCTCCCTGGAGAT	148	55
*slc2a1*	00000036904	GATCGGCTCCCTTCAGTTCG	ATGGTGGTGCTAGGGATGTTC	123	55
*socs3a*	00000018425	CAATCAACCCTGTGCGGTCA	CGATACTGGCACTGGTACGG	134	55
*stk17al*	00000007969	CAGCCAAGGCGAATCCCTAT	TCGTCGTTATCGGCAACACA	127	55
*tnfaip3*	00000017004	AAGAGGTTGAACTGGTGCCG	CAGCAGGTACTGAGACGCC	99	50
*tnfrsf18*	00000030388	GGTGGCCTGTTTAGCAGTCA	TGCACGGTGGTTTGCTTATTTT	110	non amplified

Four candidate reference genes were used to test their stability (*rplp0*, *α-tubulin, rpl8*, *18S*). The Reference Gene Selection Tool from Bio-Rad CFX Maestro software (Bio-Rad), based on geNorm software principles (63, 64) with an algorithm to normalize the Cq of each gene against the Cqs of all reference genes tested, was used to analyze the stability of reference genes. To test the expression stability of four candidate reference genes and select the best reference genes for the data normalization in our study, 16 randomly selected samples were used (representing 4 biological replicates for each group – gynogenetic infected, gynogenetic non-infected, sexual infected, and sexual non-infected). The amplification was performed under the qPCR conditions described below. The best reference genes were selected on the basis of M – value, a measure of stability for a reference gene. Genes with M values < 0.5 (*rplp0*, *α-tubulin*) were considered as stable and ideal, these reference genes were used for expression data normalization in our data set. Genes with M values between 0.5 – 1 were also revealed as acceptable (*rpl8*, *18S*). See **Supplementary Table 2** for stability of reference genes.

The PCR reaction mix (10 ul) contained 1x PCR buffer, 1.5 mM MgCl_2_, 10 mM dNTPs, 0.2 µM both forward and reverse primer, 1 U recombinant Taq DNA polymerase (Biogen), and 1 µl of prepared cDNA (see above). PCR was run under the following conditions: initial denaturation at 95°C/4 min; 30 cycles of 95°C/30 s, an optimizing annealing temperature (50/55/60°C)/30 s, 72°C/45 s; and a final amplification at 72°C/10 min.

The following qPCR was applied for each selected gene and was run on a CFX96 Real-Time PCR Detection System with a C1000 thermal cycler (Bio-Rad). Each PCR reaction (total volume 12 µl) contained 6 µl of Power SYBER Green PCR Master mix (Thermo Fisher Scientific), 0.4 µl of each primer (to a final concentration 0.33 µM of each primer in the reaction), 0.2 µl of dH_2_O, and 5 µl of cDNA template. The efficiency of amplification was checked. It varied in acceptable values for analyzed genes (E = 90 – 110%, R^2^ > 0.98, slope = -3.58 – -3.10). The amplification efficiency (E, in %), regression coefficient (R^2^), slope and y-intercept (y-int) were calculated automatically by the Bior-Rad CFX Manager 3.0. The PCR was run under the following conditions: an initial denaturation at 95°C for 10 min, followed by 40 cycles of 95°C for 15 s and a gene-specific annealing temperature (AT, included in **Table 1**) for 30 s, followed by 45 s at 72°C. A melting curve analysis was performed to verify the PCR specificity (95°C for 15 s, gene-specific AT for 1 min, and gene-specific AT to 95°C gradually increasing by 0.5°C per 5 s). The relative quantification was analyzed by Bio-Rad CFX Manager 3.0 and Bio-Rad CFX Maestro software (Bio-Rad). Using *α-tubulin* and *rplp0* as the reference genes, the relative expression values of the differentially expressed target genes, i.e. the normalized expressions, were calculated using the 2^−ΔΔCt^ method (65). A Pearson correlation was calculated between the log_2_(fold change) values from RNAseq and the log_2_(fold change) values from qPCR.

## 3 Results

### 3.1 Parasite infection in sexual and gynogenetic specimens

**Figure 1:**
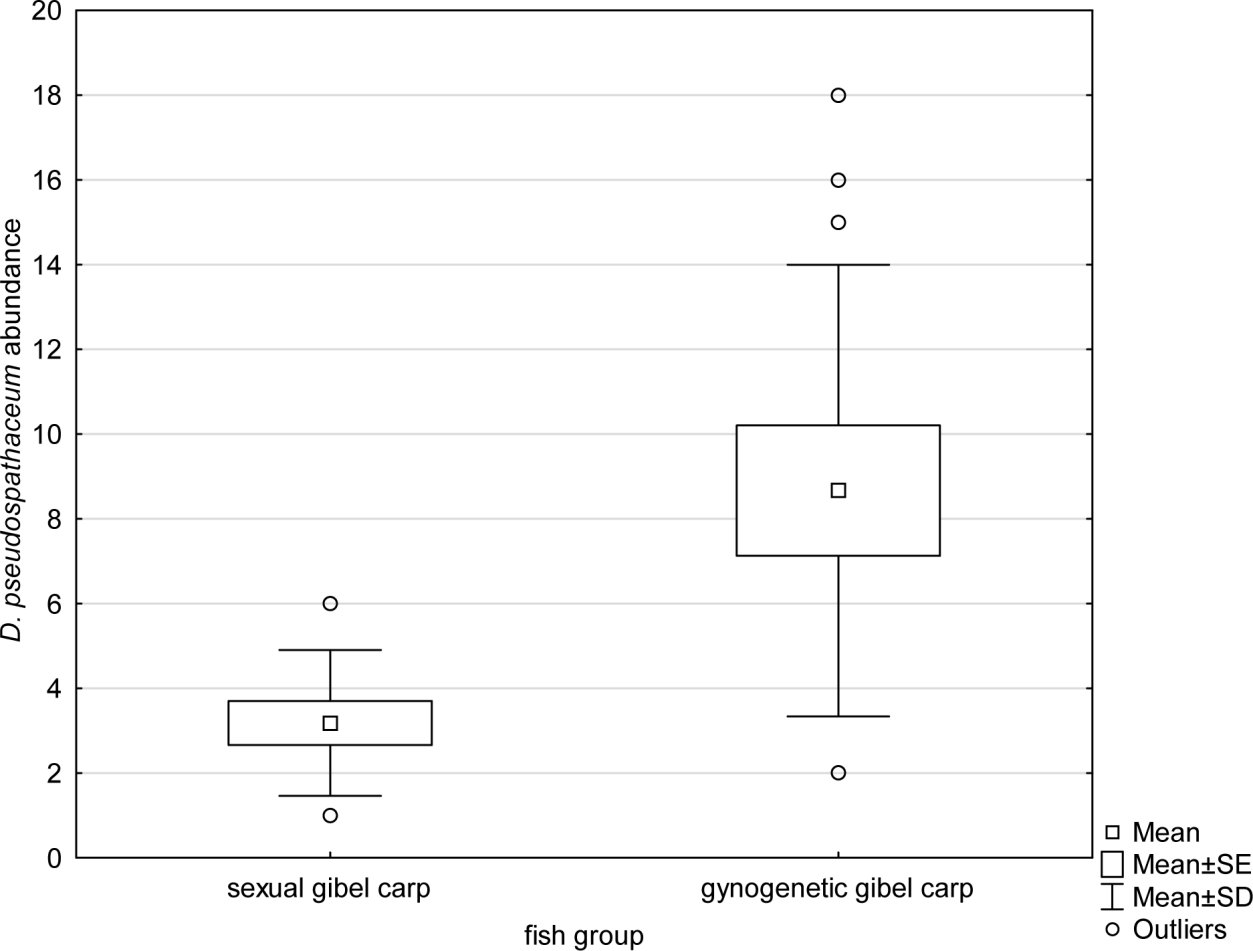
Abundance of trematode *Diplostomum pseudospathaceum* in gynogenetic and sexual gibel carp.

The intensity of infection of *Diplostomum pseudospathaceum* in sexual gibel carp ranged from 1 to 6, whilst two to 18 parasites per fish were found in gynogenetic gibel carp. A statistically significant difference was found in parasite abundance (t-test for unequal variance, t = 3.523; p = 0.003) and in intensity of infection (t = 3.255; p = 0.005) between sexual and gynogenetic gibel carp, with the gynogenetic form being more infected than the sexual one (**Figure 1**).

### 3.2 Differentially expressed genes

Principal component analysis (PCA) based on transcriptome wide gene expression (**Supplementary Figure 1**) well differentiated between infected and non-infected sexual diploid specimens and there was also the trend of differentiation between infected and non-infected gynogenetic triploid specimens. However, the variability was observed mostly in infected gynogenetic triploid specimens varying in parasite load (see above).

**Figure 2:**
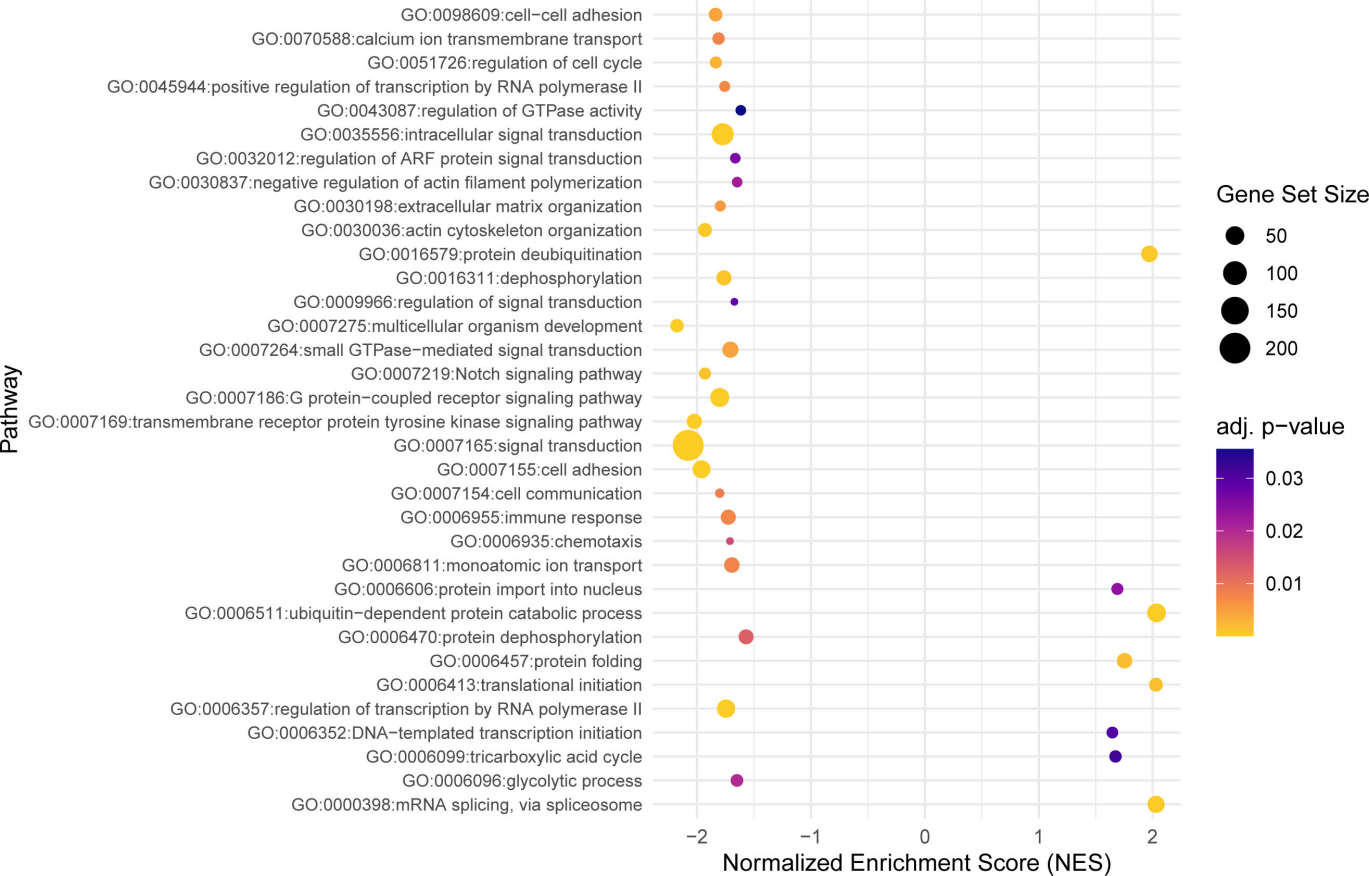
The dot plot visualizes the outcomes of the gene set enrichment analysis for the biological processes category, mapping the Normalized Enrichment Score (NES) of each pathway along the x-axis, while pathways are enumerated along the y-axis. Each dot represents a distinct pathway, with the dot’s color indicating the adjusted p-value—providing a measure of the statistical significance of the enrichment. The size of each dot correlates with the number of genes within the respective pathway, thus offering a visual representation of pathway size relative to its enrichment significance.

**Figure 3:**
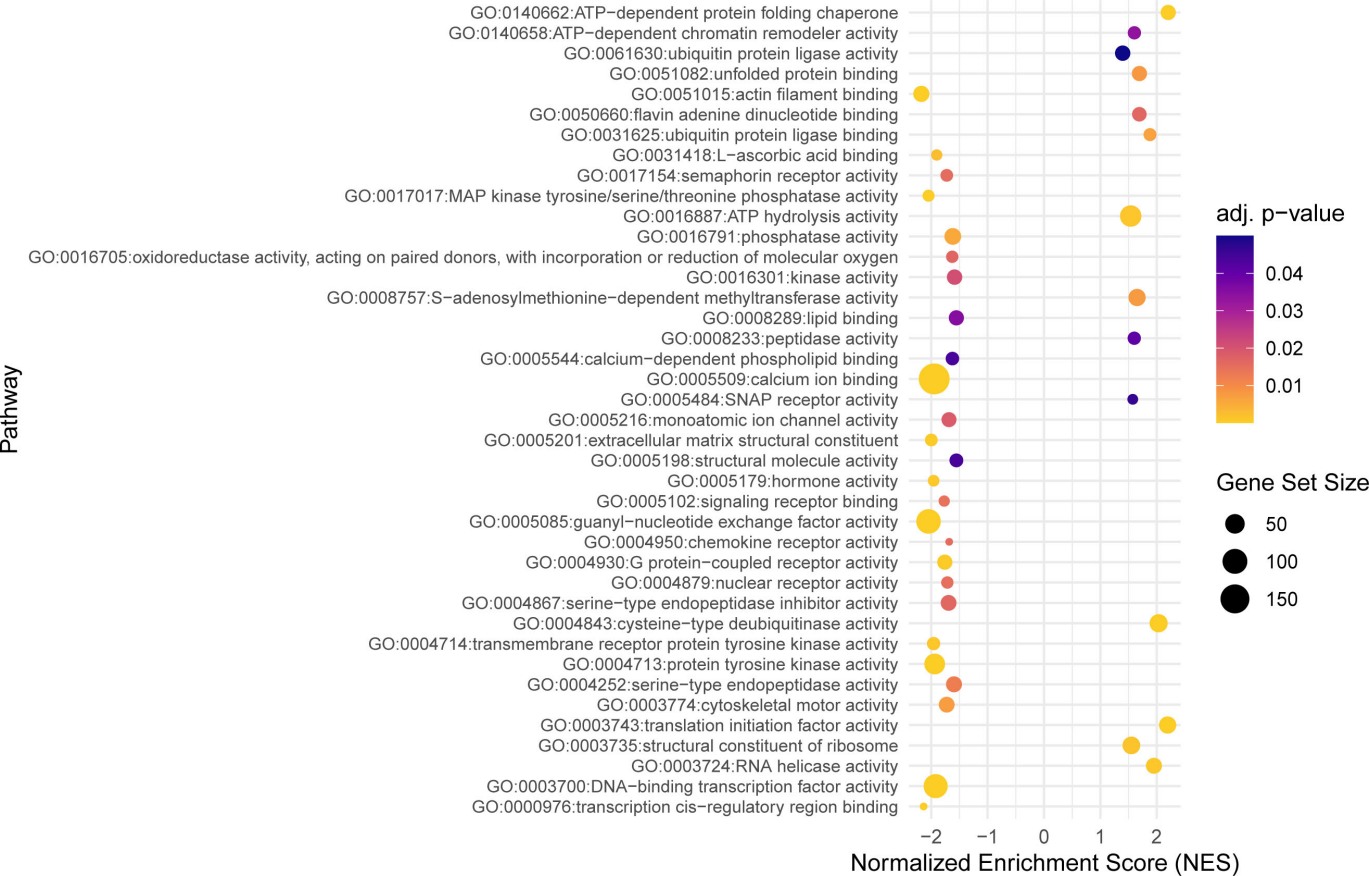
The dot plot visualizes the outcomes of the gene set enrichment analysis for the molecular functions category, mapping the Normalized Enrichment Score (NES) of each pathway along the x-axis, while pathways are enumerated along the y-axis. Each dot represents a distinct pathway, with the dot’s color indicating the adjusted p-value—providing a measure of the statistical significance of the enrichment. The size of each dot correlates with the number of genes within the respective pathway, thus offering a visual representation of pathway size relative to its enrichment significance.

**Figure 4:**
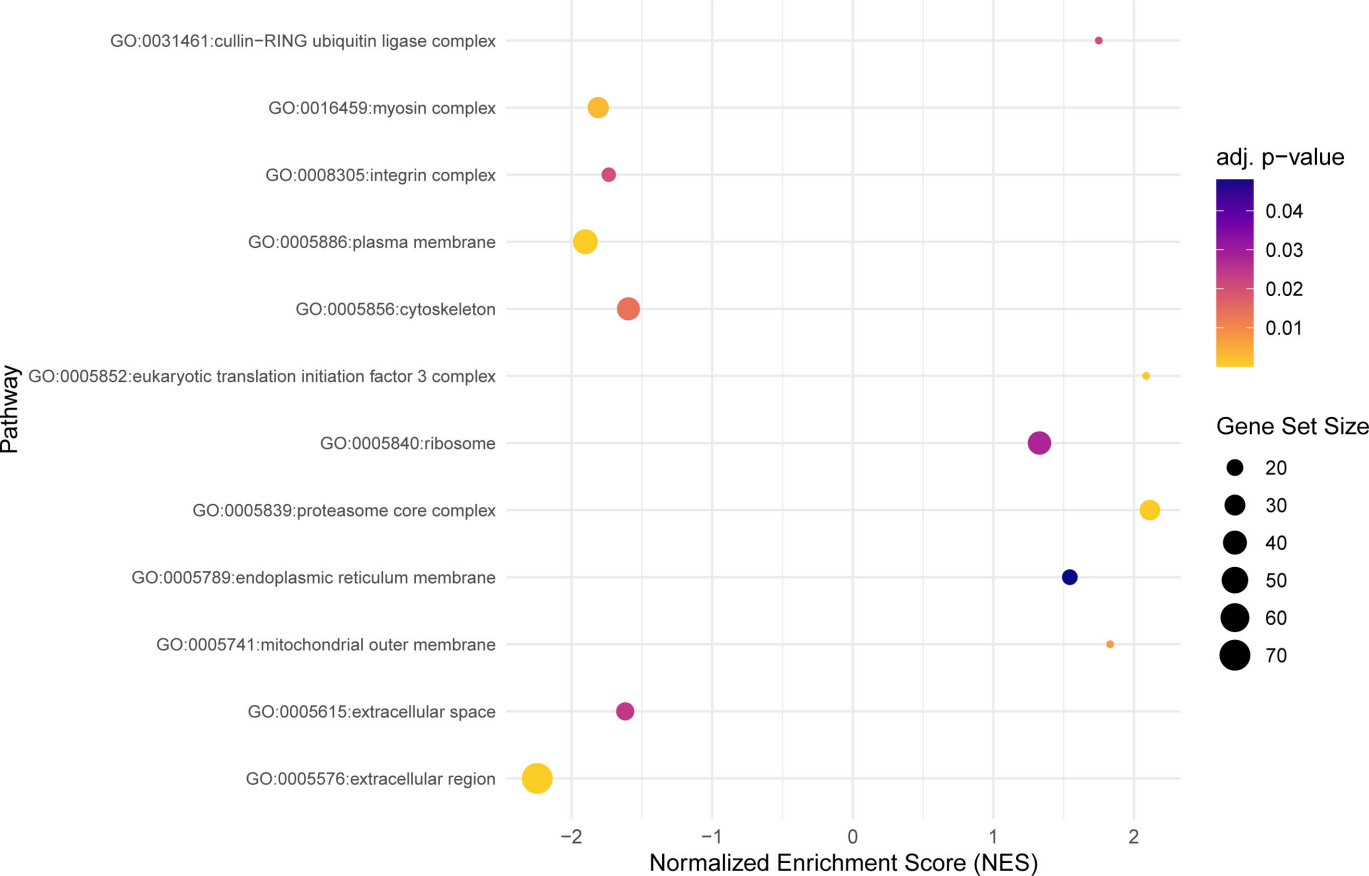
The dot plot visualizes the outcomes of the gene set enrichment analysis in the cellular components category, mapping the Normalized Enrichment Score (NES) of each pathway along the x-axis, while pathways are enumerated along the y-axis. Each dot represents a distinct pathway, with the dot’s color indicating the adjusted p-value—providing a measure of the statistical significance of the enrichment. The size of each dot correlates with the number of genes within the respective pathway, thus offering a visual representation of pathway size relative to its enrichment significance.

Using the model with interaction, a total of 302 significant DEGs were identified (P_adj_ ≤ 0.05) (**Supplementary Table 3**). Taking into account the difference between gynogenetic and sexual gibel carp, 246 genes were upregulated and 56 were downregulated in infected sexual gibel carp when compared to non-infected sexual gibel carp, whilst 260 genes were downregulated and 42 genes were upregulated in infected gynogenetic gibel carp when compared to non-infected gibel carp. The list of the genes for which a potential link to vertebrate host immunity was identified according to searches using Ensembl, Zfin and GeneCards is included in **Supplementary Table 4**. The potential functions of DEGs were determined using GO approach in terms of biological processes, molecular functions, and cellular components. Within these three main functional categories, DEGs were classified into 111 GO terms grouped into several sub-categories (below referred as groups, see **Figures 2**–**4**).

**Figure 5:**
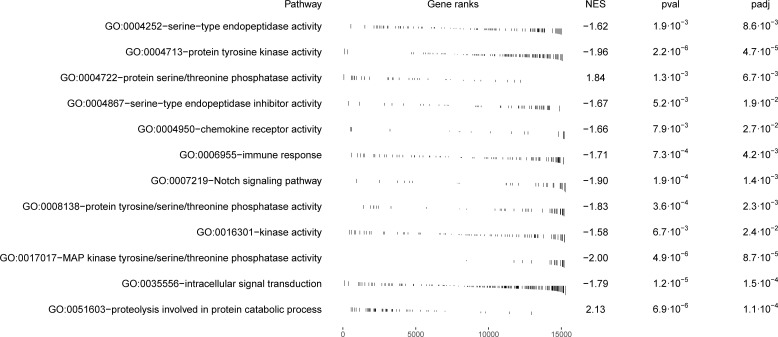
The enrichment of the 12 selected GO terms examined for the presence of immunity-associated genes. Y-axis represents pathways and the x-axis represents the enrichment of each gene within those pathways. The degree of enrichment is indicated by a normalized enrichment score (NES).

Within the biological process category, 34 top GO terms are shown in **Figure 2**. Among these GO terms, immune function (GO:0006955), Notch signaling pathway (GO:0007219) and intracellular signal transduction (GO:0035556) were examined for the presence of genes potentially associated with the parasite infection (**Figure 5**). Taking into account the difference between gynogenetic and sexual gibel carp, the significant genes selected by the model were the following: *tlr5a* and *il6* for immune function, *notch1b* and *dll4* for Notch signaling pathway, and *rgs9a*, *socs1a*, *socs3a*, *wsb1*, *net1*, *rps6ka3b* and *plce1* for intracellular signal transduction. When immune function was examined using model without reproduction mode effect, the following immune genes were revealed within immune function (GO:0006955): *il6*, *cxcl8b.3*, *c7b*, *c8b*, *c9*, *csf3a*, and *vtnb*.

Within the molecular function category, 40 top GO terms are shown in **Figure 3**. Several GO terms were examined for the presence of immune genes (**Figure 5**) and the following GO terms with corresponding genes potentially related to immunity were revealed: GO:0017017 MAP kinase tyrosine/threonine/phosphatase activity (*dusp1*, *dusp4*, *dusp5* and *dusp6*), GO:0004950 chemokine receptor activity (*ccr9a* and *cxcr4b*), GO:0004252 serine type endopeptidase activity (*prss23*), GO:0004713 protein tyrosine kinase activity (*matk* and *flt1*), GO:0008138 protein tyrosine serine threonine phosphatase activity (*ssha2*, *dusp1*, *dusp4*, *dusp5* and *dusp6*), and GO:0016301 kinase activity (*ip6k2a*, *dgkd* and *itpkcb*) (**Figure 5**).

Within the cellular components category, 12 significant GO terms were found (**Figure 4**). The cellular component category depicts several regions of the cell where most of the DEGs are expressed. The differentially expressed genes were mostly localized in the extracellular region (GO:0005576), plasma membrane (GO:0005886), the proteasome core complex (GO:0005839), and myosin complex (GO:0016459).

### 3.3 KEGG Pathway enrichment analysis of DEGs potentially associated with immune response

**Figure 6:**
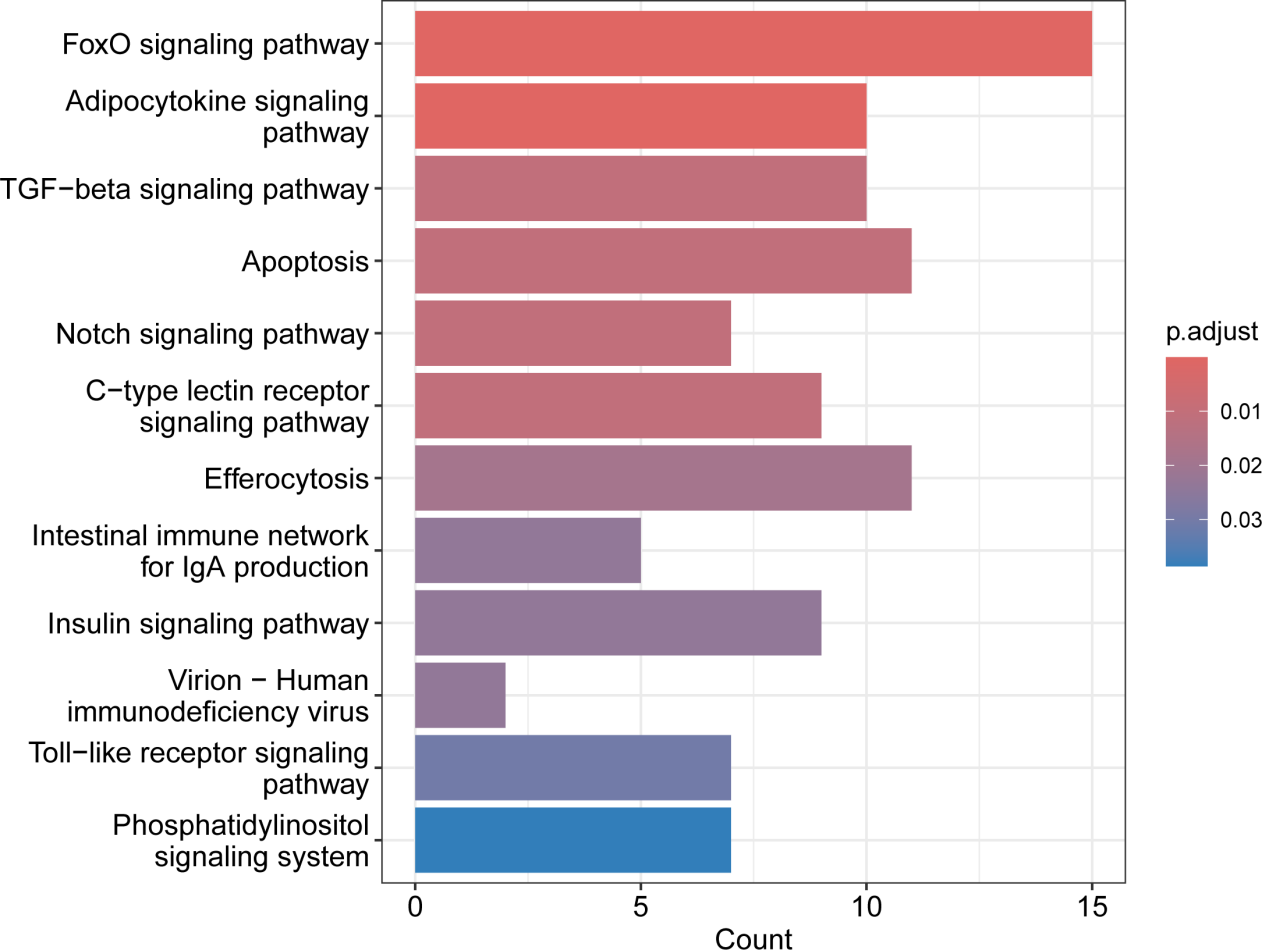
The 12 significant immunity-associated pathways selected by KEGG Pathway enrichment analysis. Gen counts for each pathway are shown on x-axis.

All DEGs (P_adj_ ≤ 0.05) were mapped to the KEGG database. A total of 52 original genes or gene isoforms were revealed corresponding to the 12 significant pathways and are shown in **Figure 6**. Whilst most of the genes were involved in a single pathway, 13 genes (or gene isoforms) were involved in multiple pathways (ranging from two to six pathways) (**Supplementary Table 5**).

Four pathways were identified within the ecological information processing category, representing the signal transduction subcategory – specifically, the FoxO signaling pathway (caua04068), the TGF-beta signaling pathway (caua04350), the Notch signaling pathway (caua04330), and the phosphatidylinositol signaling system (caua04070). Apoptosis (caua04210) was a single pathway representing the cellular processes category and the cell growth and death subcategory. The virion-human immunodeficiency virus (caua03260) was a single pathway representing the genetic information processing category and information processing in viruses. Five pathways were identified within the organismal systems category. Three of them were classified within the immune system subcategory (C - type lectin receptor signaling pathway (caua04625), the intestinal immune network for IgA production (caua04672), and the Toll-like receptor signaling pathway (caua04620)) and two of them were classified within the endocrine system subcategory (the adipocytokine signaling pathway (caua04920), and the insulin signaling pathway (caua04910)). Among the 12 significant pathways, the FoxO signaling pathway and adipocytokine signaling pathway were the most significant. The FoxO signaling pathway, apoptosis, and efferocytosis represented the pathways with the highest gene counts (**Figure 6**). The genes associated with the 12 significant pathways are shown in **Supplementary Table 5**, presented in individual fish by heatmap in **Supplementary Figure 2** and their placement within the eighth selected pathways is shown in **Supplementary Figures 3**-**10**. All these genes were revealed as important for immune response or were immunity-associated (see **Supplementary Table 4** for their function). Several genes were involved in multiple pathways, most of those genes having a function in two or three pathways, whilst *il6* and *nfkbia* were involved each in four pathways and *pik3r1* was involved in six pathways.

### 3.4 Experimental corroboration of RNAseq outputs by qPCR

**Figure 7:**
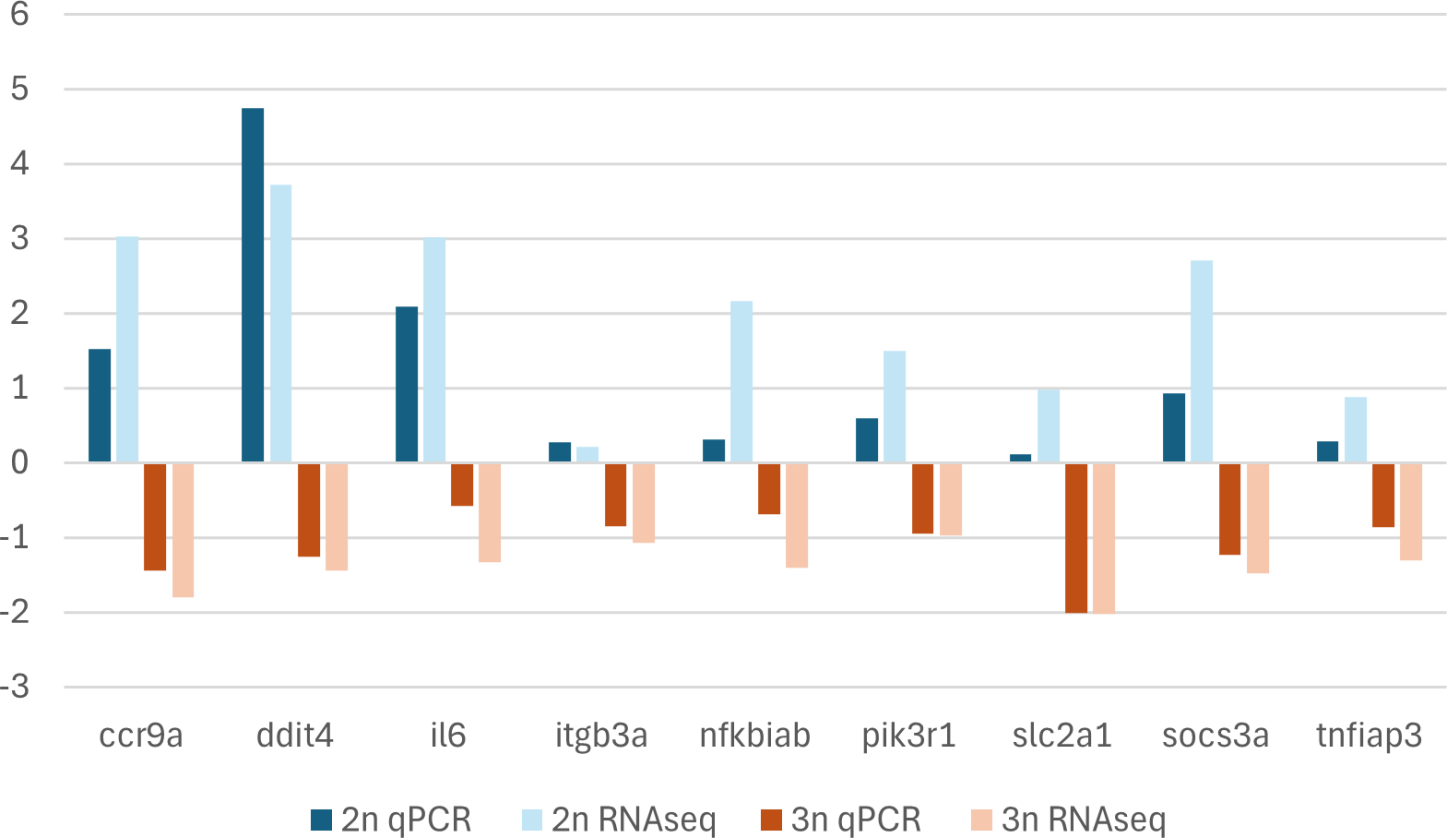
Log_2_(fold change) values for RNAseq and log_2_(fold change) values for qPCR data. The x-axis displays the target gene names. The y-axis displays the log_2_(fold change) values of the gene expression between infected and non-infected (control fish). A positive log_2_(fold change) value of the gene expression indicates that the gene was upregulated in infected fish when compared to non-infected fish. A negative log_2_(fold change) value indicates that the gene was downregulated in infected fish when compared to non-infected fish.

Eleven immunity-related and differentially expressed genes were selected for experimental corroboration by qPCR (**Table 1**). All these genes were upregulated in infected sexual gibel carp when compared to non-infected sexual gibel carp and downregulated in infected gynogenetic gibel carp when compared to non-infected gynogenetic gibel carp using RNAseq. However, two of them, specifically, *map3k8* and *stk17a* did not show the upregulation in infected diploid sexual specimens when compared to non-infected specimens using qPCR, these two genes were discarded from graphical presentation (**Figure 7**). The log_2_(fold change) values from RNAseq was positively correlated with the log2(fold change) values from qPCR using whole data set, i.e. including sexual diploid and gynogenetic triploid specimens (R = 0.898, p < 0.001), as well as when analyzing sexual diploid group and gynogenetic triploid group separately (R = 0.786, p = 0.012 for sexual diploid group, and R = 0.799, p = 0.011 using for gynogenetic triploid group). Log_2_(fold change) values from qPCR and log_2_(fold change) values from RNAseq for sexual diploids and gynogenetic triploids are plotted in **Figure 7**.

## 4 Discussion

Our study focused on infection by mixed genotypes of the eye fluke *Diplostomum pseudospathaceum* and the analysis of the transcriptome profile to reveal expressed genes differentially between infected and non-infected specimens of gynogenetic and sexual gibel carp. In accordance with the Red Queen hypothesis, higher parasite load was found in clonal fish when compared to genetically diverse sexual fish. Transcriptome profile analysis revealed a set of genes expressed differentially after parasite infection. Surprisingly, however, the majority of these genes showed opposite trends of regulation in gynogenetic and sexual gibel carp, which may potentially indicate the loss of capacity in clonally reproducing fish to induce an effective immune response.

Differences in the susceptibility to parasites and the varying effectiveness of immune response between the sexual and gynogenetic gibel carp were proposed and investigated as the potential mechanisms behind their co-occurrence in the same habitats (19, 24). Both studies investigated gibel carp for the presence of naturally occurring metazoan parasites, mostly represented by ectoparasitic monogeneans. The study by Šimková et al. (19) included analyses of the MHC profiles of gynogenetic and sexual gibel carp and revealed that gynogens with the most common MHC genotypes were more parasitized when compared to sexuals. This was interpreted to be in line with the Red Queen hypothesis, i.e., if asexual reproduction occurs in nature and the asexual clone becomes the most common genotype in a host population, co-evolutionary interactions with parasites (parasites are under selection to infect the most common host genotype in a local host population) may favor sexual reproduction in hosts because of high genetic variability (25, 26). However, Pakosta et al. (24) analyzed the parasite load in gynogenetic and sexual gibel carp without considering the individual MHC genotype, in four consecutive years, and revealed strong interannual variation but no effect of reproduction strategy on parasite load, which may likely be explained by possible genetic variation among clone lineages. In contrast, the higher susceptibility of gynogenetic triploid females of Japanese crucian carp (*Carassius auratus langsdorfii*) when compared to sexual diploid females was shown even with respect to the trematode *Metagonimus* sp. from Japan (17), a parasite with a complex life cycle penetrating the skin of a fresh or brackish water fish (which is in the role of second intermediate host) and encysting as metacercariae in the tissue. The study of Hakoyama et al. (17) showed the lower baseline immune response in the gynogenetic form, suggesting the lower immune activity of the phagocytes (nonspecific immune reaction) potentially explaining higher trematode load.

As *Diplostomum* metacercariae are exposed to the immune system only for a short period of time before reaching and developing in the immunologically-protected eye lens of the fish (24 hours), only the innate immune mechanisms should act immediately and effectively before encystations (44, 66). However, the role of MHC genes representing the adaptive immune system was investigated also in the case of *D. pseudospathaceum* parasitizing three-spined sticklebacks (*Gasterosteus aculeatus*), with the expectation that adaptive immunity is upregulated when challenged with infection (67). Their study revealed that MHC class IIB expression is increased by approximately 50% in fish lines selected for higher parasite load (i.e., fish lines having a low innate response). Finally, Noreikiene et al. (68) performed eye transcriptome analysis and revealed the overexpression of immune-related genes and the presence of the eye diplostomid parasite *Tylodelphys clavata* in Eurasian perch (*Perca fluviatilis*) living in clear-water lakes, highlighting the necessity to reevaluate the traditional view of the eye as an “immune-privileged” organ. Thus, in our study, we performed the experiment using the same dose of metacercariae of *D. pseudospathaceum* per individual immune-naïve gibel carp and revealed that the parasite is more successful in reaching the target organ, i.e. the eye lens, in gynogenetic fish then in the sexuals. This finding may indicate that gynogenetic fish are either more susceptible to free-living cercariae penetrating fish skin, or alternatively that both gynogenetic and sexual fish are susceptible to cercariae but that the immune response of sexuals is more effective in eliminating the parasite in the course of its migration to the eye lens. Using the transcriptome profile of the spleen we revealed the differential expression of immunity-related genes in gibel carp differing between gynogens and sexuals of gibel carp. Our results indicate that studying the molecular mechanisms of innate vs. adaptive immunity using the transcriptomics of skin representing the point of entrance of a diplostomid parasite into the fish body could help us understand the pattern observed from spleen transcriptomics. Using the GO of DEGs, we revealed some GO terms directly or indirectly associated with immune response. The immune response GO term belonging to the biological processes category refers to any immune system process that functions in the calibrated response of an organism to a potential internal or invasive threat (69). However, there are also other biological processes that are involved in host immune response, like those corresponding to the GO terms intracellular signal transduction and signal transduction, as immune response depends on intracellular signaling events (70). In our study, the genes involved in signal transduction (i.e. *pik3r1*, *socs3a*) were also involved in immune response. DEGs corresponding to immune response and other genes associated with immunity inferred from GO analyses were individually discussed on the basis of their KEGG designation within corresponding pathways (see below), and 11 of these genes were successfully validated by the qPCR approach. On the basis of KEGG analyses, the successfully annotated DEGs represented 12 significant pathways related to host immunity.

The FoxO signaling pathway is involved in many cellular physiological events such as apoptosis, cell-cycle control, glucose metabolism, oxidative stress resistance, and longevity. The role of the FoxO signaling pathway has already been documented in fish; this pathway was enriched in both spleen and head kidney of largemouth bass (*Micropterus salmoides*) after infection by *Aeromonas hydrophila*, with phosphatidylinositol 3-kinase (*pik3r3b*) and serine/threonine-protein kinase (*plk2*) being among four target genes. The down-regulated expression of so-called cell death related genes, including *plk2* among others, and the up-regulated expression of inflammation related genes, including *il6* among others, was shown in the hybrid grouper *Epinephelus fuscoguttatus* x *Epinephelus lanceolatus* after bacterial infection (71). Enriched genes within the FoxO signaling pathway (specifically, *pi3k*, *sgk1*, *foxo1*, *prmt1*, *pcka*) were revealed by KEGG analysis in gynogenetic blunt snout bream (*Megalobrama amblycephala*) in a study exploring the molecular mechanisms underlying enhanced hypoxia tolerance as a result of gynogenesis (72). In our study, we documented the importance of 15 genes within the FoxO signaling pathway, including also five genes identified in target fish organs under parasite infection or other stressors by previous studies (see above), specifically *plk2*, *plk3, sgk1*, *pik3r1a* (phosphatidylinositol 3-kinase regulatory subunit alpha-like) and *il6*. All the genes identified within the FoxO signaling pathway were downregulated in gynogenetic females of gibel carp but upregulated in sexuals of the same species.

Adipocyte-derived bioactive substances, i.e. adipocytokines (or adipokines), generally play a significant role in the self-defense system against metabolic overload. However, adipose tissue is increasingly seen as playing an important role in immune inflammation and immune response (73, 74). In rainbow trout (*Oncorhynchus mykiss*), Pignatelli et al. (75) showed that adipose tissue produces different immune mediators and identified the presence of B lymphocytes expressing IgM, IgD or IgT, CD8a+ cells, and cells expressing MHC-II. They also showed the modulation of a wide range of secreted immune factors within adipose tissue in response to viral infection (VHSV). Up-regulation of the genes enriched within the adipocytokine signaling pathway was found after nervous necrosis virus infection in medaka (*Oryzias latipes*) (76). The role of the adipocytokine signaling pathway was found to play a significant role in high-salinity stress in spotted scat (*Scatophagus argus*), with upregulation of the following genes: *g6pc1*, *socs1*, *socs3*, *adipor2*, *pck1*, and *ppara* in liver of fish treated in high salinity conditions (77). Our study revealed ten differentially expressed genes (or isoforms) with opposite directions of regulation in gynogenetic and sexual fish after *D. pseudospathaceum* infection, with some of them (especially *nfkbia*, *g6pc1*, *socs3*) already revealed by previous studies [e.g (74–77)]. Several DEGs enriched within the adipocytokine signaling pathway belonging to endocrine system in our study of gibel carp were also reported within the next endocrine system pathway – specifically, the insulin signaling pathway (*socs3a*, *g6pc*, *g6pc1a.2*, *irs2a* and *irs2b*). The upregulation of solute carrier genes (*slc*) was also shown in fish in relation to salinity-related stress (78). Among multiple genes revealed from the transcriptomic study of head kidney and gills, the role of the cytokine signaling-associated gene *socs3* and leukocyte-related gene *slc3a2* in the immune response of *Gasterosteus aculeatus* infected by *D. pseudospathaceum* was revealed by Haase et al. (45). Similarly, our study revealed the importance of *socs1*, *socs3a*, and *slc2a1b* (the last one also enriched within efferocytosis) in establishing immune response in gibel carp infected by eye trematode.

The TGF-beta signaling pathway has important regulatory functions in animal growth, development, wound repair, bone metabolism, and immunity. In our study of gibel carp, *smad* (1, 6 and 7), *lrrc32*, *grem1*, and *skilb* classified within this pathway were found to be differentially regulated after fish were infected by *D. pseudospathaceum*. *Smads* were previously found to be important TGFβ1 signaling pathway genes involved in the regulation of the growth and immunity of crucian carp (*Carassius carassius*) (79), and affecting embryonic development in rainbow trout (*Oncorhynchus mykiss*) (80). The role of several genes involved in the TGF-beta signaling pathway (having, however, rather marginal importance for fish immunity) was also revealed in a study of goldfish (*C. auratus*) when elucidating the immune mechanisms against the monogenean parasite *Gyrodactylus kobayashii* and using the transcriptome profiles of goldfish skin (81). The role of *lrrc32* in platelet-platelet and platelet-endothelial cell interactions, and the promotion of thrombus formation was suggested from functional genomics in zebrafish (82); however, *lrrc32* which encodes glycoprotein A repetitions predominant (GARP), was established as a candidate disease-associated gene in humans [i.e (83, 84)]. Similarly, the role of *grem1* (encoding gremlin1) was not studied in fish; however, the overexpression of *grem1* was shown to be associated with mostly inflammatory disease in mammals [e.g (85, 86)]. The DEGs within the Notch signaling pathway (*notch1b* and *dll4*) were also inferred from our study. Both the TGF-beta and Notch signaling pathways together with the other eight signaling pathways were significant when investigating the skin immune response of zebrafish (*Danio rerio*) to *Aeromonas hydrophila* infection (87). The eye trematode *D. pseudospathaceum* enters the fish body by penetrating the skin; however, the molecular mechanisms associated with innate immunity at the early stage of its infection and during migration to the eyes are still unknown.

Apoptosis is one of the principal strategies of host cells to clear pathogens and maintain organismal homeostasis. The enhanced expression of several genes whose roles were revealed within the apoptosis pathway in the present study of gibel carp was previously found in Nile tilapia (*Oreochromis niloticus*) infected by tilapia lake virus; these genes were, specifically, *nfkbia* within the nfkbia pathway, and *socs* within the JAK-STAT pathway, suggesting that the virus was able to successfully establish infection by subverting the host’s immune response. The roles of other genes revealed in our study, specifically pro-apoptosis genes (such as DNA damage-inducible transcript 3 (*ddit3*), and growth arrest and DNA damage-inducible alpha (*gadd45a*)), and the TNF receptor superfamily member were enriched in mammals after *Toxoplasma gondii* infection (88). Concerning fish, upregulation of the genes associated with apoptosis was also found in relation to high temperature stress (such as the *casp* and *trail* genes), suggesting functional hypoxia (89) or the downregulation of some genes in relation to viral infection [e.g. *casp8* in the study of Xiang et al. (90)]. In our study, we revealed the upregulation of *cflar*, also called *c-flip* (CASP8 and FADD-like apoptosis regulator), and *tnfrsf1a* (tumor necrosis factor receptor superfamily member 1A-like) together with *gadd45a* in sexual gibel carp after *D. pseudospathaceum* infection, likely indicating the potential of sexuals to establish an immune reaction to parasite-induced stress, and/or, alternatively, the insufficient capacity of gynogenetic fish to establish an appropriate cell response to trematode infection. Concerning the role of efferocytosis in host immunity, it is considered to be an effective means of clearing apoptotic cells by professional and non-professional phagocytes, the efferocytosis is associated with the pathogenesis of various inflammatory disorders. Even if the differential expression of genes involved in this pathway was not previously investigated/recognized in parasitized fish, efferocytosis was documented as a mechanism for eliminating bacteria, parasites, viruses, and pathogen-infected cells in mammals (91). However, the expression of genes belonging to the integrin beta superfamily (*itg* genes), playing an essential role in intercellular connection and signal transmission, and *slc* genes (see above) was previously found to be modified by infection by a specific *D. pseudospathaceum* lineage (45). The role of these genes was also revealed in our study. In other cyprinids, specifically in common carp (*C. carpio*), the low expression of *itgβ1* was documented in fish resistant to viral infection (CyHV3) (92). In addition, other genes revealed in our study to be involved in gibel carp innate immunity when infected by *D. pseudospathaceum* were previously found to change their expression under the effects of pathogens or other stressors in fish – specifically, *dusp1* [e.g. Podok et al. (93)], *hif* [e.g. Shi et al. (94)], *gpr* [e.g. Kaczorek et al. (95)], and *cebpb* [e.g. Rojo et al. (96)].

Concerning the enriched DEGs directly involved in host immunity when gibel carp is infected by *D. pseudospathaceum*, they belong to three KEGG pathways (the C-type lectin receptor signaling pathway, the intestinal immune network for IgA production, and the Toll-like receptor signaling pathway) assigned within the immune system of the organismal systems category. These three pathways have previously been shown to be among 20 pathways of DEGs enriched in the transcriptomics of head kidney of black carp (*Mylopharyngodon piceus*) infected by grass carp reovirus (GCRV), highlighting the role of antiviral innate immunity (97). In our study, the important immune genes revealed by KEGG analyses within immune system pathways included *nfkbia*, *il6*, *cxcr4*, *ccr9* (and *ccr9a*), *tlr5a*, *mapk8*, as well as genes also involved in other immunity-related pathways, i.e. *plk3* (involved also in FoxO signaling pathway) and *pik3r1a* (involved in 6 from 12 top KEGG pathways). In addition to immune system pathways, *cxcr4* (C-X-C chemokine receptor type 4-B-like) was also assigned within the subcategory termed information processing in viruses belonging to the genetic information processing. The *cxcr4* having a role in antiviral response was found in gibel carp throughout the MAPK3 and JAK/STAT pathways (98). A study of orange-spotted grouper (*Epinephelus coioides*) by Lin et al. (99) suggested that *cxcr4* may not only play a role in the early immune response to microbial infection but also inhibits the immune system and central nervous system. The upregulation of *ccr7*, *ccr9*, *cxcr3B* and *cxcr4* was previously shown in rainbow trout (*Oncorhynchus mykiss*) after infection by VHSV (100). The differential expression of immune genes in relation to *D. pseudospathaceum* infection using its different clones was previously investigated in three-spined sticklebacks; surprisingly, only six genes were jointly upregulated in head kidney, and other up- or down-regulated DEGs differed among fish infected by specific clones of *D. pseudospathaceum* (45). Among up-regulated genes, their study revealed the function of *ccr9* [a chemokine receptor interacting with its ligand TECK, which attracts dendritic cells and macrophages (101)] in sticklebacks infected by a specific *D. pseudospathaceum* clone. This was explained by the fact that innate immune gene responses in vertebrates can be specific to different parasite genotypes. However, our study also highlights the role of the hosts (sexual vs. gynogenetic), as *ccr9* and *cxcr4* were among many DEGs that were up-regulated in sexual gibel carp and down-regulated in gynogenetic gibel carp. Our study clearly evidences that host genotype influences the parasite infection rate and suggests the disadvantage faced by clonally reproducing fish from the point of views of parasite infection and immunity response to parasite infection. Haase et al. (45) indicated that the complement system activated via different pathways is a central part of the fish response to *D. pseudospathaceum*. Later, Haase et al. (48) investigated the effects of homologous and heterologous exposures to genetically-distinct lineages of *D. pseudospathaceum* on the gene expression patterns of adaptive immunity in sticklebacks and showed distinct expression patterns in the heterologous pre-exposed fish specimens. Haase et al. (48) revealed again the importance of the complement system in both affecting innate immunity as well as mediating adaptive responses. In our model applied to select DEGs having different effects in sexual and gynogenetic fish, the genes associated with the complement system were not significant; however, their differential expression across the whole sample of infected (by mixed genotypes of *D. pseudospathaceum*) and non-infected gibel carp was evidenced using model without reproduction mode effect (i.e. selecting the immunity-associated DEGs acting in the same direction in gynogenetic triploids and sexual diploids).

Two genes – *tlr5* and *il6*, as two important markers of innate immunity - were involved in gibel carp immune response when fish were infected by *D. pseudospathaceum*, the first one through the Toll-like receptor signaling pathway, the second one through all three immune pathways (the C-type lectin receptor signaling pathway, the intestinal immune network for IgA production, and the Toll-like receptor signaling pathway) and the immunity-associated FoxO signaling pathway (see above). The role of *tlr5* in fish immunity was previously shown in relation to bacterial infection, which is because of its capacity to recognize bacterial flagellin in hosts. The expression of this gene plays a critical role in resisting bacterial infection and an auxiliary regulatory role in early infection. The down- or upregulation of *tlr5* was found depending on the target organ and pathogen [i.e (102–104)]. The *il6* gene encodes interleukin 6, which is a member of Th2 cytokines released by APCs. This gene plays a significant role in inflammation and autoimmunity, and a critical role in B cell maturation into IgG secreting cells. There is wide evidence of the role of *il6* expression in fish affected by pathogen infection, this gene acted through the various immunity-associated pathways [i.e (105–107)]; however, challenge experiments with the eyeworm *Oxyspirura petrowi* in Northern bobwhite (*Colinus virginianus*) also revealed changes in the expression of genes encoding cytokines and Toll-like receptors including *il6*. Finally, the enrichment of DEGs belonging to MAPK (mitogen-activated protein kinase), nuclear factor-kappa B (NF-kappa B), and the Toll-like receptor (TLR) pathways was documented in fish infected by bacteria (108) and protozoan ciliate (109). Some of the genes associated with these pathways, even if evidenced as enriched within other immunity-associated pathways in our study, seem to play an important role in fish immune response to infection by the eye trematode *D. pseudospathaceum*.

## 5 Conclusion

The present study revealed the difference in infection rate by trematode *Diplostomum pseudospathaceum* between gynogenetic and sexual fish hosts, this supporting the Red Queen hypothesis, which postulates the greater susceptibility of clonal hosts to parasite infection when compared to genetically various sexual hosts. We performed transcriptome profile analysis of an immune organ (here, the spleen) in gibel carp infected by *D. pseudospathaceum*, and revealed that the eye parasite alters the gene expression. Specifically, we showed the differential gene expression induced by eye parasite infection to have various impacts on gynogenetic and sexual hosts, documenting for the majority of DEGs upregulation in sexuals, and downregulation in asexuals, which may suggest the limited potential of asexuals to cope with higher parasite infection, and which may represent some destabilization of the asexual genome. Our study implies that parasite-mediated selection and the modulation of organismal responses to parasite infection (measured at the level of transcriptome profile changes between infected and non-infected fish) – these varying between asexuals and sexuals, especially with respect to immune response regulation – represent the key mechanisms contributing to the coexistence of phylogenetically and ecologically related asexual and sexual forms in natural habitats. However, we should also consider the fact that certain loci or even whole genomes (generally, as documented in allopolyploids) are up- or downregulated (or even silenced) as ploidy increases (110, 111). As gynogenetic gibel carp is triploid, the down-regulation of DEGs related to *D. pseudospathaceum* infection may be explained by this phenomenon, which will require further molecular investigation in the future.

In spite of the fact that metacercariae of *D. pseudospathaceum* are situated in an immune-privileged organ, we showed that the eye parasite may provoke the host’s immune response, which may also be partially related to the migration route, starting with penetration of the skin and finishing with localization in the eye. However, we emphasize that the expression of immune genes in asexual and sexual hosts may vary depending on the specific role of the gene in an organism and on the intensity and course of the infection.

## Data availability statement

The datasets presented in this study can be found in online repositories. The data used in this study have been deposited in NCBI’s Gene Expression Omnibus and are accessible through GEO Series accession number GSE256052 (https://www.ncbi.nlm.nih.gov/geo/query/acc.cgi?acc=GSE256052).

## Ethics statement

The experiment was approved by the Animal Care and Use Committee at the Faculty of Science, Masaryk University in Brno (Czech Republic). The study was conducted under the experimental project approved by the Ministry of Education, Youth and Sports of the Czech Republic under document n. 30071/2022–5.

## Author contributions

MF: Investigation, Writing – review & editing. TT: Formal analysis, Methodology, Data curation, Visualization, Writing – review & editing. MO: Investigation, Methodology, Resources, Writing – review & editing. KCK: Methodology, Validation, Writing – review & editing. MS: Methodology, Validation, Writing – review & editing. KV: Writing – review & editing, Methodology, Validation. MD: Formal analysis, Methodology, Writing – review & editing. LV: Methodology, Resources, Writing – review & editing. AŠ: Conceptualization, Project administration, Formal analysis, Funding acquisition, Investigation, Supervision, Visualization, Writing – original draft, Writing – review & editing.

## References

[1] Lusková V Lusk S Halačka K Vetešník L. *Carassius auratus gibelio* – the most successful invasive fish in waters of the Czech Republic. Russ J Biol Invasions. (2010) 1:176–80. doi: 10.1134/S2075111710030069

[2] Fuad MMH Vetešník L Šimková A. Is gynogenetic reproduction in gibel carp (*Carassius gibelio*) a major trait responsible for invasiveness? J Vertebr Biol. (2021) 70:21049. doi: 10.25225/jvb.21049

[3] Tobler M Schlupp I. Parasites in sexual and asexual mollies (*Poecilia*, Poeciliidae, Teleostei): a case for the Red Queen? Biol Lett. (2005) 1:166–68. doi: 10.1098/rsbl.2005.0305 PMC1626213 17148156

[4] Neaves WB Baumann P. Unisexual reproduction among vertebrates. Trends Genet. (2011) 27:81–8. doi: 10.1016/j.tig.2010.12.002 21334090

[5] Lusková V Halačka K Vetešník L Lusk S. Changes of ploidy and sexuality status of “*Carassius auratus*” populations in the drainage area of the River Dyje (Czech Republic). Ecohydrol Hydrobiol. (2004) 4:165–71.

[6] Przybył A Przybylski M Spóz A Juchno D Szabelska A Kowalewska K. Sex, size and ploidy ratios of *Carassius gibelio* from Poland. Aquat Invasions. (2020) 15:335–54. doi: 10.3391/ai.2020.15.2.08

[7] Paschos I Nathanailides C Tsoumani M Perdikaris C Gouva E Leonardos I. Intra and inter-specific mating options for gynogenetic reproduction of *Carassius gibelio* (Bloch, 1783) in Lake Pamvotis (NW Greece). Belg J Zool. (2004) 134:55–60.

[8] Barbuti R Mautner S Carnevale G Milazzo P Rama A Sturmbauer C. Population dynamics with a mixed type of sexual and asexual reproduction in a fluctuating environment. BMC Evol Biol. (2012) 12:49. doi: 10.1186/1471-2148-12-49 22489797 PMC3353185

[9] Mee JA Rowe L. A comparison of parasite loads on asexual and sexual *Phoxinus* (Pisces: Cyprinidae). Can J Zool. (2006) 84:808–16. doi: 10.1139/z06-064

[10] Mee JA Taylor EB. The cybrid invasion: widespread postglacial dispersal by *Phoxinus* (Pisces: Cyprinidae) cytoplasmic hybrids. Can J Zool. (2012) 90:577–84. doi: 10.1139/z2012-023

[11] Lamatsch DK Schmid M Schartl M. A somatic mosaic of the gynogenetic Amazon molly. J Fish Biol. (2002) 60:1417–22. doi: 10.1111/j.1095-8649.2002.tb02436.x

[12] Lamatsch DK Lampert KP Fischer P Geiger M Schlupp I Schartl M. Diploid Amazon mollies (*Poecilia formosa*) show a higher fitness than triploids in clonal competition experiments. Evol Ecol. (2009) 23:687–97. doi: 10.1007/s10682-008-9264-2

[13] Tobler M Schlupp I. Differential susceptibility to food stress in neonates of sexual and asexual mollies (*Poecilia*, Poeciliidae). Evol Ecol. (2010) 24:39–47. doi: 10.1007/s10682-008-9288-7

[14] Schlupp I. Mate choice and the amazon molly: how sexuality and unisexuality can coexist. J Hered. (2010) 101:S55–61. doi: 10.1093/jhered/esq015 20421327

[15] Janko K Eisner J. Sperm-dependent parthenogens delay the spatial expansion of their sexual hosts. J Theor Biol. (2009) 261:431–40. doi: 10.1016/j.jtbi.2009.08.012 19698721

[16] Choleva L Apostolou A Ráb P Janko K. Making it on their own: sperm-dependent hybrid fishes (*Cobitis*) switch the sexual hosts and expand beyond the ranges of their original sperm donors. Philos Trans R Soc B Biol Sci. (2008) 363:2911–19. doi: 10.1098/rstb.2008.0059 PMC2606746 18508748

[17] Hakoyama H Nishimura T Matsubara N Iguchi K. Difference in parasite load and nonspecific immune reaction between sexual and gynogenetic forms of *Carassius auratus* . Biol J Linn Soc. (2001) 72:401–7. doi: 10.1111/j.1095-8312.2001.tb01326.x

[18] Hakoyama H Iwasa Y. Coexistence of a sexual and an unisexual form stabilized by parasites. J Theor Biol. (2004) 226:186–94. doi: 10.1016/j.jtbi.2003.08.012 14643188

[19] Šimková A Košař M Vetešník L Vyskočilová M. MHC genes and parasitism in *Carassius gibelio*, a diploid-triploid fish species with dual reproduction strategies. BMC Evol Biol. (2013) 13:122. doi: 10.1186/1471-2148-13-122 23768177 PMC3691641

[20] Šimková A Hyršl P Halačka K Vetešník L. Physiological and condition-related traits in the gynogenetic-sexual *Carassius auratus* complex: different investments promoting the coexistence of two reproductive forms? BMC Evol Biol. (2015) 15:154. doi: 10.1186/s12862-015-0438-6 26245328 PMC4545816

[21] Vetešník L Lusk S Halačka K Spurný P. Morphometric characteristics and growth of *Carassius auratus* in the lower part of the River Dyje (Czech Republic). Ecohydrol Hydrobiol. (2004) 4:215–21.

[22] Vetešník L Halačka K Šimková A. The effect of ploidy and temporal changes in the biochemical profile of gibel carp (*Carassius gibelio*): a cyprinid fish species with dual reproductive strategies. Fish Physiol Biochem. (2013) 39:171–80. doi: 10.1007/s10695-012-9688-z 22773226

[23] Xie J Wen JJ Chen B Gui JF. Differential gene expression in fully-grown oocytes between gynogenetic and gonochoristic crucian carps. Gene. (2001) 271:109–16. doi: 10.1016/s0378-1119(01)00491-7 11410372

[24] Pakosta T Vetešník L Šimková A. A long temporal study of parasitism in asexualsexual populations of *Carassius gibelio*: does the parasite infection support coevolutionary Red Queen dynamics? BioMed Res Int. (2018) 2018:6983740. doi: 10.1155/2018/6983740 29713645 PMC5866858

[25] Hamilton WD. Sex versus non-sex versus parasite. Oikos. (1980) 35:282–90. doi: 10.2307/3544435

[26] Hamilton WD Axelrod R Tanese R. Sexual reproduction as an adaptation to resist parasites (a review). Proc Natl Acad Sci U S A. (1990) 87:3566–73. doi: 10.1073/pnas.87.9.3566 PMC53943 2185476

[27] Lively CM Craddock C Vrijenhoek RC. Red Queen hypothesis supported by parasitism in sexual and clonal fish. Nature. (1990) 344:864–67. doi: 10.1038/344864a0

[28] Weeks SC. A reevaluation of the Red Queen model for the maintenance of sex in a clonal-sexual fish complex (Poeciliidae: *Poeciliopsis*). Can J Fish Aquat Sci. (1996) 53:1157–64. doi: 10.1139/f96-041

[29] Scharsack JP Kalbe M. Differences in susceptibility and immune responses of three-spined sticklebacks (*Gasterosteus aculeatus*) from lake and river ecotypes to sequential infections with the eye fluke *Diplostomum pseudospathaceum* . Parasit Vectors. (2014) 7:109. doi: 10.1186/1756-3305-7-109 24656136 PMC3994412

[30] Niewiadomska K. Verification of the life-cycles of *Diplostomum spathaceum* (Rudolphi, 1819) and D. pseudospathaceum Niewiadomska, 1984 (Trematoda, Diplostomidae). Syst Parasitol. (1986) 8:23–31. doi: 10.1007/BF00010306

[31] Frey RA Barrett LM Parkin L Blakeley B Alund M Byford G. Eye flukes (*Diplostomum* spp.) damage retinal tissue and may cause a regenerative response in wild threespine stickleback fish. Exp Eye Res. (2022) 225:109298. doi: 10.1016/j.exer.2022.109298 36288754 PMC13285514

[32] Hakalahti T Karvonen A Valtonen ET. Climate warming and disease risks in temperate regions - *Argulus coregoni* and *Diplostomum spathaceum* as case studies. J Helminthol. (2006) 80:93–8. doi: 10.1079/joh2006351 16768854

[33] Palmieri JR Cali A Heckmann RA. Experimental biological-control of eye fluke, *Diplostomum spathaceum*, by a protozoan hyperparasite, *Nosema strigeoidae* (Protozoa, Microsporida). J Parasitol. (1976) 62:325–26. doi: 10.2307/3279300 1263047

[34] Michálková V Ondračková M. Experimental evidence for parasite-induced over-winter mortality in juvenile. Rhodeus amarus. J Fish Biol. (2014) 84:1377–88. doi: 10.1111/jfb.12363 24773537

[35] Crowden AE Broom DM. Effects of the eyefluke, *Diplostomum spathaceum*, on the behaviour of dace (*Leuciscus leuciscus*). Anim Behav. (1980) 28:287–94. doi: 10.1016/S0003-3472(80)80031-5

[36] Mikheev VN Pasternak AF Taskinen J Valtonen ET. Parasite-induced aggression and impaired contest ability in a fish host. Parasit Vectors. (2010) 3:17. doi: 10.1186/1756-3305-3-17 20226098 PMC2845576

[37] Seppälä O Karvonen A Valtonen ET. Parasite-induced change in host behaviour and susceptibility to predation in an eye fluke - fish interaction. Anim Behav. (2004) 68:257–63. doi: 10.1016/j.anbehav.2003.10.021

[38] Seppälä O Karvonen A Valtonen ET. Manipulation of fish host by eye flukes in relation to cataract formation and parasite infectivity. Anim Behav. (2005) 70:889–94. doi: 10.1016/j.anbehav.2005.01.020

[39] Mikheev VN Pasternak AF. Structure of aggressive behavior in underyearlings of the rainbow trout *Oncorhynchus mykiss* (Salmonidae) changes under the influence of *Diplostomum pseudospathaceum* (Trematoda) parasites. J Ichthyol. (2023) 63:816–21. doi: 10.1134/S0032945223040136

[40] Nezhybová V Reichard M Methling C Ondračková M. Limited impacts of chronic eye fluke infection on the reproductive success of a fish host. Biol J Linn Soc. (2020) 129:334–46. doi: 10.1093/biolinnean/blz189

[41] Jakobsson S Brick O Kullberg C. Escalated fighting behavior incurs increased predation risk. Anim Behav. (1995) 49:235–39. doi: 10.1016/0003-3472(95)80172-3

[42] McDougall PT Kramer DL. Short-term behavioral consequences of territory relocation in a Caribbean damselfish, *Stegastes diencaeus* . Behav Ecol. (2007) 18:53–61. doi: 10.1093/beheco/arl055

[43] Buchmann K. Antiparasitic immune mechanisms in teleost fish: a two-edged sword? Bull Eur Ass Fish Pathol. (2000) 20:48–59.

[44] Rauch G Kalbe M Reusch TBH. One day is enough: rapid and specific host–parasite interactions in a stickleback-trematode system. Biol Lett. (2006) 2:382–84. doi: 10.1098/rsbl.2006.0462 PMC1686187 17148409

[45] Haase D Rieger JK Witten A Stoll M Bornberg-Bauer E Kalbe M. Specific gene expression responses to parasite genotypes reveal redundancy of innate immunity in vertebrates. PLoS One. (2014) 9:e108001. doi: 10.1371/journal.pone.0108001 25254967 PMC4177871

[46] Whyte SK Chappell LH Secombes CJ. Cytotoxic reactions of rainbow trout, *Salmo gairdneri* Richardson, macrophages for larvae of the eye fluke *Diplostomum spathaceum* (Digenea). J Fish Biol. (1989) 35:333–45. doi: 10.1111/j.1095-8649.1989.tb02986.x

[47] Woo PTK. Immunological responses of fish to parasitic organisms. Ann Rev Fish Dis. (1992) 2:339–66. doi: 10.1016/0169-4758(87)90178-5

[48] Haase D Rieger JK Witten A Stoll M Bornberg-Bauer E Kalbe M. Immunity comes first: The effect of parasite genotypes on adaptive immunity and immunization in three-spined sticklebacks. Dev Comp Immunol. (2016) 54:137e144. doi: 10.1016/j.dci.2015.09.008 26400836

[49] Karvonen A Hudson PJ Seppälä O Valtonen ET. Transmission dynamics of a trematode parasite: exposure, acquired resistance and parasite aggregation. Parasitol Res. (2004) 92:183–88. doi: 10.1007/s00436-003-1035-y 14652746

[50] Georgieva S Soldánová M Pérez-del-Olmo A Dangel RD Sitko J Sures B. Molecular prospecting for European *Diplostomum* (Digenea: Diplostomidae) reveals cryptic diversity. Int J Parasit. (2013) 43:57–72. doi: 10.1016/j.ijpara.2012.10.019 23201234

[51] Bush AO Lafferty KD Lotz JM Shostaket AW. Parasitology meets ecology on its own terms: Margolis et al. revisited. J Parasitol. (1997) 83:575–83. doi: 10.2307/3284227 9267395

[52] Andrews S. FastQC: A Quality Control Tool for High Throughput Sequence Data. (2010). Available online at: http://www.bioinformatics.babraham.ac.uk/projects/fastqc/.

[53] Bushnell B. BBMap: A fast, accurate, splice-aware aligner. Lawrence Berkeley National Laboratory. LBNL Report #: LBNL-7065E. (2014). Available at: https://escholarship.org/uc/item/1h3515gn

[54] Dobin A Carrie A Davis CA Schlesinger F Drenkow J Zaleski C. STAR: ultrafast universal RNA-seq aligner. Bioinformatics. (2013) 29:15–21. doi: 10.1093/bioinformatics/bts635 23104886 PMC3530905

[55] Liao Y Smyth GK Shi W. FeatureCounts: an efficient general purpose program for assigning sequence reads to genomic features. Bioinformatics. (2014) 30:923–30. doi: 10.1093/bioinformatics/btt656 24227677

[56] R Core Team. A language and environment for statistical computing. Vienna, Austria: R Foundation for Statistical Computing (2023). Available at: https://www.R-project.org/.

[57] Love MI Huber W Anders S. Moderated estimation of fold change and dispersion for RNA-seq data with DESeq2. Genom Biol. (2014) 15:550. doi: 10.1186/s13059-014-0550-8 PMC4302049 25516281

[58] Benjamini Y Hochberg Y. Controlling the false discovery rate: a practical and powerful approach to multiple testing. J R Stat Soc Ser B. (1995) 57:289–300. doi: 10.1111/j.2517-6161.1995.tb02031.x

[59] Korotkevich G Sukhov V Budin N Shpak B Artyomov MN Sergushichev A. Fast gene set enrichment analysis. bioRxiv. (2021). doi: 10.1101/060012

[60] Supek F Bošnjak M Škunca N Šmuc T. REVIGO summarizes and visualizes long lists of gene ontology terms. PLoS One. (2011) 6:7. doi: 10.1371/journal.pone.0021800 PMC3138752 21789182

[61] Durinck S Spellman P Birney E Huber W. Mapping identifiers for the integration of genomic datasets with the R/Bioconductor package biomaRt. Nat Protoc. (2009) 4:1184–91. doi: 10.1038/nprot.2009.97 PMC3159387 19617889

[62] Yu G Wang L Han Y He Q. ClusterProfiler: an R package for comparing biological themes among gene clusters. OMICS. (2012) 16:284–7. doi: 10.1089/omi.2011.0118 PMC3339379 22455463

[63] Pfaffl MW. A new mathematical model for relative quantification in real-time RT-PCR. Nucleic Acids Res. (2001) 29:e45. doi: 10.1093/nar/29.9.e45 11328886 PMC55695

[64] Vandesompele J De Preter K Pattyn F Poppe B Van Roy N De Paepe A. Accurate normalization of real-time quantitative RT-PCR data by geometric averaging of multiple internal control genes. Genome Biol. (2002) 3:1–12. doi: 10.1186/gb-2002-3-7-research0034 PMC126239 12184808

[65] Livak KJ Schmittgen TD. Analysis of relative gene expression data using real - time quantitative PCR and the 2–ΔΔCT method. Methods. (2001) 25:402–8. doi: 10.1006/meth.2001.1262 11846609

[66] Kalbe M Kurtz J. Local differences in immunocompetence reflect resistance of sticklebacks against the eye fluke *Diplostomum pseudospathaceum* . Parasitology. (2005) 132:1–12. doi: 10.1017/S0031182005008681 16393359

[67] Wegner KM Kalbe M Reusch TBH. Innate versus adaptive immunity in sticklebacks: evidence for trade-offs from a selection experiment. Evol Ecol. (2007) 21:473–83. doi: 10.1007/s10682-006-9129-5

[68] Noreikiene K Ozerov M Ahmad F Kõiv T Kahar S Gross R. Humic- acid-driven escape from eye parasites revealed by RNA-seq and target-specific metabarcoding. Parasit Vectors. (2020) 13:433. doi: 10.1186/s13071-020-04306-9 32859251 PMC7456052

[69] Carbon S Ireland A Mungall CJ Shu SQ Marshall B Lewis S. AmiGO: online access to ontology and annotation data. Bioinformatics. (2009) 25:288–9. doi: 10.1093/bioinformatics/btn615 PMC2639003 19033274

[70] Neagu M Constantin C. Signal transduction in immune cells and protein kinases. Adv Exp Med Biol. (2021) 1275:133–49. doi: 10.1007/978-3-030-49844-3_5 33539014

[71] He L Zhao L Li Q Huang L Qin Y Zhuang Z. *Pseudomonas plecoglossicida* fliP gene affects the immune response of *Epinephelus fuscoguttatus* ♀×*Epinephelus lanceolatus* ♂ to infection. Fish Shellfish Immunol. (2023) 140:108971. doi: 10.1016/j.fsi.2023.108971 37481102

[72] Gong D Xu L Li W Shang R Chen J Hu F. Comparative analysis of liver transcriptomes associated with hypoxia tolerance in the gynogenetic blunt snout bream. Aquaculture. (2020) 523:735163. doi: 10.1016/j.aquaculture.2020.735163

[73] Sauerwein H Bendixen E Restelli L Ceciliani F. The adipose tissue in farm animals: a proteomic approach. Curr Protein Pept Sci. (2014) 15:146–55. doi: 10.2174/1389203715666140221123105 24555890

[74] Sun J Bian CC Ji SH Luo XL Ji H. Greater potency of adipocytes compared with preadipocytes under lipopolysaccharide exposure in grass carp *Ctenopharyngodon idella* . Fish Shellfish Immunol. (2019) 91:343–49. doi: 10.1016/j.fsi.2019.04.295 31042574

[75] Pignatelli J Castro R Granja AG Abós B González L Jensen LB. Immunological characterization of the teleost adipose tissue and its modulation in response to viral infection and fat-content in the diet. PLoS One. (2014) 9:e110920. doi: 10.1371/journal.pone.0110920 25333488 PMC4204996

[76] Wang YD Rajanbabu V Chen JY. Transcriptome analysis of medaka following epinecidin-1 and TH1–5 treatment of NNV infection. Fish Shellfish Immunol. (2015) 42:121–31. doi: 10.1016/j.fsi.2014.10.040 25449377

[77] Chen J Cai B Tian C Jiang D Shi H Huang Y. RNA sequencing (RNA-Seq) analysis reveals liver lipid metabolism divergent adaptive response to low- and high-salinity stress in spotted scat (*Scatophagus argus*). Animals. (2023) 13:1503. doi: 10.3390/ani13091503 37174540 PMC10177406

[78] Jiang JL Xu J Ye L Sun ML Jiang ZQ Mao MG. Identification of differentially expressed genes in gills of tiger puffer (*Takifugu rubripes*) in response to low-salinity stress. Comp Biochem Physiol B. (2020) 243–4:110437. doi: 10.1016/j.cbpb.2020.110437 32247057

[79] Li C Wang L. Molecular characterization, expression and functional analysis of TGFβ1-b in crucian carp (*Carassius carassius*). Int J Biol Macromol. (2020) 165:1392–401. doi: 10.1016/j.ijbiomac.2020.10.024 33045298

[80] Gahr SA Weber GM Rexroad CE. Identification and expression of Smads associated with TGF-b/activin/nodal signaling pathways in the rainbow trout (*Oncorhynchus mykiss*). Fish Physiol Biochem. (2012) 38:1233–44. doi: 10.1007/s10695-012-9611-7 22290475

[81] Zhou S Liu Y Dong J Yang Q Xu N Yang Y. Transcriptome analysis of goldfish (*Carassius auratus*) in response to *Gyrodactylus kobayashii* infection. Parasitol Res. (2021) 120:161–71. doi: 10.1007/s00436-020-06827-9 33094386

[82] O’Connor MN Salles II Cvejic A Watkins NA Walker A Garner SF. Functional genomics in zebrafish permits rapid characterization of novel platelet membrane proteins. Blood. (2009) 113:4754–62. doi: 10.1182/blood-2008-06-162693 PMC2680375 19109564

[83] Harel T Levy-Lahad E Daana M Mechoulam H Horowitz-Cederboim S Gur M. Homozygous stop-gain variant in LRRC32, encoding a TGFβ receptor, associated with cleft palate, proliferative retinopathy, and developmental delay. Eur J Hum Genet. (2019) 27:1315–9. doi: 10.1038/s41431-019-0380-y PMC6777458 30976112

[84] Lehmkuhl P Gentz M Garcia de Otezya AC Grimbacher B Schulze-Koops H Skapenko A. Dysregulated immunity in PID patients with low GARP expression on Tregs due to mutations in LRRC32. Cell Mol Immunol. (2021) 18:1677–91. doi: 10.1038/s41423-021-00701-z PMC8245512 34059789

[85] Qu S Liu Z Wang B. Down-regulation of Gremlin1 inhibits inflammatory response and vascular permeability in chronic idiopathic urticaria through suppression of TGF-β signaling pathway. Gene. (2020) 756:144916. doi: 10.1016/j.gene.2020.144916 32580008

[86] Xie Z Zhou G Zhang M Han J Wang Y Li X. Recent developments on BMPs and their antagonists in inflammatory bowel diseases. Cell Death Discovery. (2023) 9:210. doi: 10.1038/s41420-023-01520-z 37391444 PMC10313712

[87] Lü AJ Hu XC Wang Y Zhu AH Shen LL Tian J. Skin immune response in the zebrafish, *Danio rerio* (Hamilton), to *Aeromonas hydrophila* infection: a transcriptional profiling approach. J Fish Dis. (2015) 38:137–50. doi: 10.1111/jfd.12214 24517469

[88] Du K Lu F Xie C Ding H Shen Y Gao Y. *Toxoplasma gondii* infection induces cell apoptosis *via* multiple pathways revealed by transcriptome analysis. J Zhejiang Univ Sci B. (2022) 23:315–27. doi: 10.1631/jzus.B2100877 PMC9002249 35403386

[89] Ma F Zhao L Ma R Wang J Du L. FoxO signaling and mitochondria-related apoptosis pathways mediate tsinling lenok trout (*Brachymystax lenok tsinlingensis*) liver injury under high temperature stress. Int J Biol Macromol. (2023) 251:126404. doi: 10.1016/j.ijbiomac.2023.126404 37597633

[90] Xiang Y Jia P Liu W Yi M Jia K. Comparative transcriptome analysis reveals the role of p53 signalling pathway during red-spotted grouper nervous necrosis virus infection in *Lateolabrax japonicus* brain cells. J Fish Dis. (2019) 42:585–95. doi: 10.1111/jfd.12960 PMC7166548 30659619

[91] Karaji N Sattentau QJ. Efferocytosis of pathogen-infected cells. Front Immunol. (2017) 22:1863. doi: 10.3389/fimmu.2017.01863 PMC5743670 29312342

[92] Jiang X Sun J Li C Hu X Ge Y Li B. Molecular cloning and sequence characterization of common carp (*Cyprinus carpio*) integrin β1 (ITGβ1) and its temporal expression in response to CyHV-3. Aquac Int. (2021) 29:1869–84. doi: 10.1007/s10499-021-00723-4

[93] Podok P Wang H Xu L Xu D Lu L. Characterization of myeloid-specific peroxidase, keratin 8, and dual specificity phosphatase 1 as innate immune genes involved in the resistance of crucian carp (*Carassius auratus gibelio*) to cyprinid herpesvirus 2 infection. Fish Shellfish Immunol. (2014) 41:531–40. doi: 10.1016/j.fsi.2014.10.001 25312688

[94] Shi X Gao F Zhao X Pei C Zhu L Zhang J. Role of HIF in fish inflammation. Fish Shellfish Immunol. (2023) 143:109222. doi: 10.1016/j.fsi.2023.109222 37956798

[95] Kaczorek E Szarek J Mikiewicz M Terech-Majewska E Schulz P Małaczewska J. Effect of feed supplementation with kynurenic acid on the morphology of the liver, kidney and gills in rainbow trout (*Oncorhynchus mykiss* Walbaum, 1792), healthy and experimentally infected with *Yersinia ruckeri* . J Fish Dis. (2017) 40:873–84. doi: 10.1111/jfd.12567 27690267

[96] Rojo I de Ilárduya ÓM Estonba A Pardo MA. Innate immune gene expression in individual zebrafish after *Listonella anguillarum.* inoculation. Fish Shellfish Immunol. (2007) 23:1285e1293. doi: 10.1016/j.fsi.2007.07.002 17804254

[97] Liu J Yan Y Yan J Wang J Wei J Xiao J. Multi-omics analysis revealed crucial genes and pathways associated with black carp antiviral innate immunity. Fish Shellfish Immunol. (2020) 106:724–32. doi: 10.1016/j.fsi.2020.08.047 32871249

[98] Lu WJ Zhou L Gao FX Zhou YL Li Z Zhang XJ. Dynamic and differential expression of duplicated cxcr4/cxcl12 genes facilitates antiviral response in hexaploid gibel carp. Front Immunol. (2020) 11:2176. doi: 10.3389/fimmu.2020.02176 33013914 PMC7516010

[99] Lin CY Chen YM Hsu HH Shiu CT Kuo HC Chen TY. Grouper (*Epinephelus coioides*) CXCR4 is expressed in response to pathogens infection and early stage of development. Dev Comp Immunol. (2012) 36:112–20. doi: 10.1016/j.dci.2011.06.009 21726578

[100] Aquilino C Castro R Fischer U Tafall C. Transcriptomic responses in rainbow trout gills upon infection with viral hemorrhagic septicemia virus (VHSV). Dev Comp Immunol. (2014) 44:12–20. doi: 10.1016/j.dci.2013.11.006 24269609

[101] Zaballos A Gutiérrez J Varona R Ardavín C Márquez G. Cutting edge: Identification of the orphan chemokine receptor GPR-9–6 as CCR9, the receptor for the chemokine TECK. J Immunol. (1999) 162:5671–5. doi: 10.4049/jimmunol.162.10.5671 10229797

[102] Liu F Su B Fu Q Shang M Gao C Tan F. Identification, characterization and expression analysis of TLR5 in the mucosal tissues of turbot (*Scophthalmus maximus* L.) following bacterial challenge. Fish Shellfish Immunol. (2017) 68:272–9. doi: 10.1016/j.fsi.2017.07.021 28705722

[103] Xue X Caballero-Solares A Hall JR Umasuthan N Kumar S Jakob E. Transcriptome profiling of atlantic salmon (*Salmo salar*) parr with higher and lower pathogen loads following *Piscirickettsia salmonis* infection. Front Immunol. (2021) 12:789465. doi: 10.3389/fimmu.2021.789465 35035387 PMC8758579

[104] Zhan F Li Y Shi F Lu Z Yang M Li Q. Characterization analysis of TLR5a and TLR5b immune response after different bacterial infection in grass carp (*Ctenopharyngodon idella*). Fish Shellfish Immunol. (2023) 136:108716. doi: 10.1016/j.fsi.2023.108716 37001745

[105] Cordero H Mauro M Cuesta A Cammarata M Esteban MA. *In vitro* cytokine profile revealed differences from dorsal and ventral skin susceptibility to pathogen-probiotic interaction in gilthead seabream. Fish Shellfish Immunol. (2016) 56:188–91. doi: 10.1016/j.fsi.2016.07.018 27422755

[106] Huang P Cai J Yu D Tang J Lu Y Wu Z. An IL-6 gene in humphead snapper (*Lutjanus sanguineus*): Identification, expression analysis and its adjuvant effects on *Vibrio harveyi* OmpW DANN vaccine. Fish Shellfish Immunol. (2019) 95:546–55. doi: 10.1016/j.fsi.2019.11.013 31704205

[107] Huang L Zhao L Liu W Xu X Su Y Qin Y. Dual RNA-seq unveils *Pseudomonas plecoglossicida htpG* gene functions during host-pathogen interactions with *Epinephelus coioides* . Front Immunol. (2019) 10:984. doi: 10.3389/fimmu.2019.00984 31130962 PMC6509204

[108] Ling X Dong W Zhang Y Qian X Zhang W He W. Comparative transcriptomics and histopathological analysis of crucian carp infection by atypical *Aeromonas salmonicida* . Fish Shellfish Immunol. (2019) 94:294–307. doi: 10.1016/j.fsi.2019.09.006 31491530

[109] Valle A Leiro JM Pereiro P Figueras A Novoa B Dirks RPH. Interactions between the parasite *Philasterides dicentrarchi* and the immune system of the turbot *Scophthalmus maximus* . A transcriptomic analysis Biol. (2020) 9:337. doi: 10.3390/biology9100337 PMC7602577 33076342

[110] King KC Seppälä O Neiman M. Is more better? Polyploidy and parasite resistance. Biol Lett. (2012) 8:598–600. doi: 10.1098/rsbl.2011.1152 22258448 PMC3391438

[111] Guo Z Liliom K Fischer DJ Bathurst IC Tomei LD Kiefer MC. Molecular cloning of a high-affinity receptor for the growth factor- like lipid mediator lysophosphatidic acid from *Xenopus* oocytes. Proc Natl Acad Sci USA. (1996) 93:14367–72. doi: 10.1073/pnas.93.25.14367 PMC26138 8962057

