# Supplementary material for: Trematode Diplostomum pseudospathaceum inducing differential immune gene expression in sexual and gynogenetic gibel carp (Carassius gibelio): parasites facilitating the coexistence of two reproductive forms of the invasive species
Source: Front Immunol. 2024 Jun 25;15:1392569. doi: 10.3389/fimmu.2024.1392569 (PMC11231671; doi:10.3389/fimmu.2024.1392569)
Supplement: Supplementary file 6 [file DataSheet_1.pdf]

Supplementary material

Supplementary table 1. Numbers of input reads, uniquely- and multi-mapped reads per sample. Ctrl – non-infected (control) fish, inf – infected fish, 2n – sexual diploid fish, 3n – gynogenetic triploid fish, m – males, f – females.

Supplementary table 2. Analysis of stability of reference genes.

Supplementary table 3. List of the differently expressed genes after *D. pseudospathaceum* infection in gynogenetic and sexual gibel carp.

Supplementary table 4. List of selected differently expressed genes revealed by Ensembl, Zfin and GeneCards databases to be involved in immune response processes. The description of gene functions according to these databases is included.

Supplementary table 5. List of the genes involved in 12 significant pathways revealed from KEGG analysis.

Supplementary figure 1. Principal component analysis (PCA) of normalized RNAseq read counts on the first two principal components (ctrl_2n – sexual diploid non-infected (control), inf_2n – sexual diploid infected, ctrl_3n – gynogenetic triploid non-infected (control), inf_3n – gynogenetic triploid infected).

Supplementary figure 2. Heatmap for individual fish specimens including the genes associated with 12 pathways selected by KEGG analysis (ctrl_2n – sexual diploid non-infected (control), inf_2n – sexual diploid infected, ctrl_3n – gynogenetic triploid non-infected (control), inf_3n – gynogenetic triploid infected).

Supplementary figure 3. KEGG map of FoxO signaling pathway. Colors indicate the significant DEGs selected using model with reproduction (gynogenetic vs. sexual) and treatment (infected vs. control).

Supplementary figure 4. KEGG map of adipocytokine signaling pathway. Colors indicate the significant DEGs selected using model with reproduction (gynogenetic vs. sexual) and treatment (infected vs. control).

Supplementary figure 5. KEGG map of insulin signaling pathway. Colors indicate the significant DEGs selected using model with reproduction (gynogenetic vs. sexual) and treatment (infected vs. control).

Supplementary figure 6. KEGG map of TGF-beta signaling pathway. Colors indicate the significant DEGs selected using model with reproduction (gynogenetic vs. sexual) and treatment (infected vs. control).

Supplementary figure 7. KEGG pathway map of apoptosis. Colors indicate the significant DEGs selected using model with reproduction (gynogenetic vs. sexual) and treatment (infected vs. control).

Supplementary figure 8. KEGG map of C-type lectin receptor signaling pathway. Colors indicate the significant DEGs selected using model with reproduction (gynogenetic vs. sexual) and treatment (infected vs. control).

Supplementary figure 9. KEGG map of Toll-like receptor signaling pathway. Colors indicate the significant DEGs selected using model with reproduction (gynogenetic vs. sexual) and treatment (infected vs. control).

Supplementary figure 10. KEGG pathway map of phosphatidylinositol signaling system. Colors indicate the significant DEGs selected using model with reproduction (gynogenetic vs. sexual) and treatment (infected vs. control).
