# Supplementary figures and images for: Trematode Diplostomum pseudospathaceum inducing differential immune gene expression in sexual and gynogenetic gibel carp (Carassius gibelio): parasites facilitating the coexistence of two reproductive forms of the invasive species
Source: Front Immunol. 2024 Jun 25;15:1392569. doi: 10.3389/fimmu.2024.1392569 (PMC11231671; doi:10.3389/fimmu.2024.1392569)

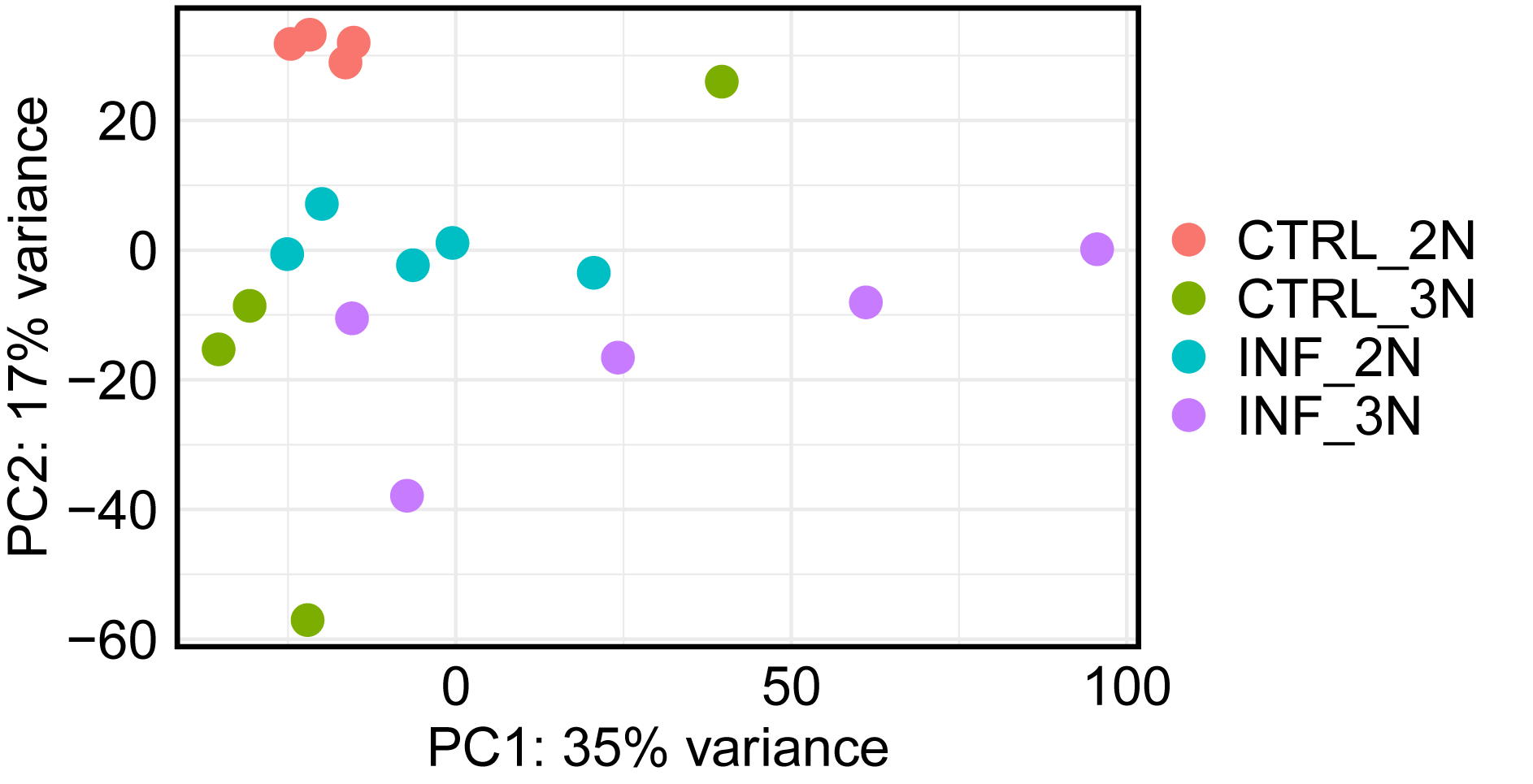

Supplement: Supplementary file 7 [file Image_1.tif]

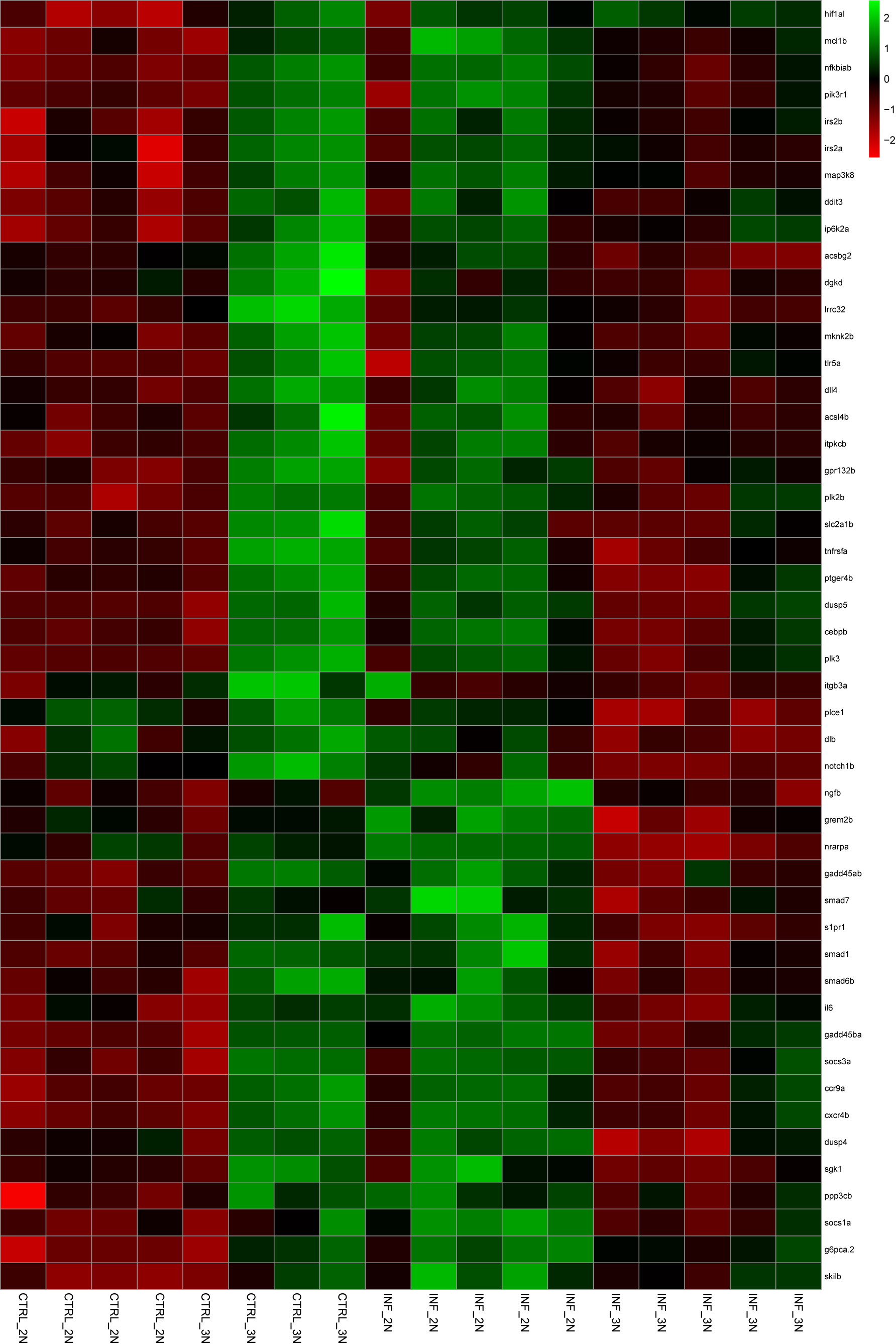

Supplement: Supplementary file 8 [file Image_2.tif]

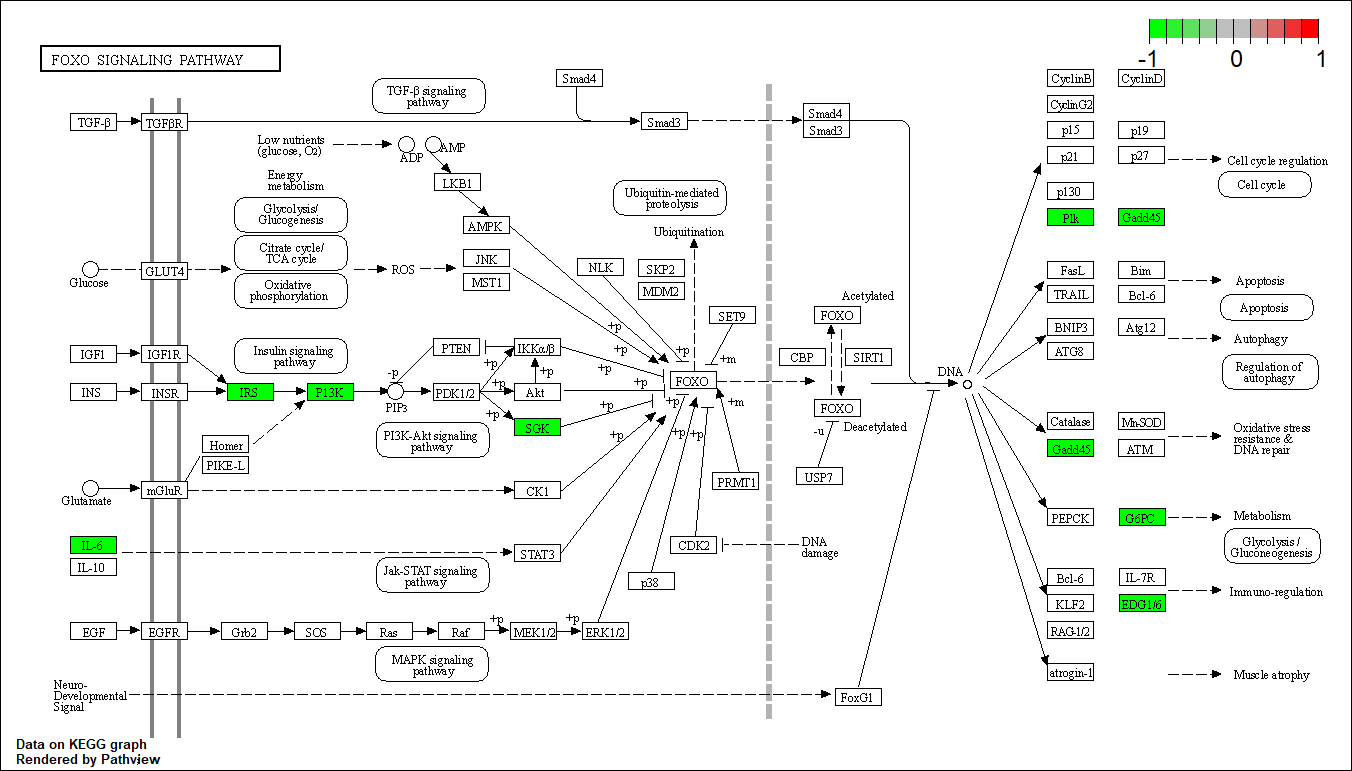

Supplement: Supplementary file 9 [file Image_3.tiff]

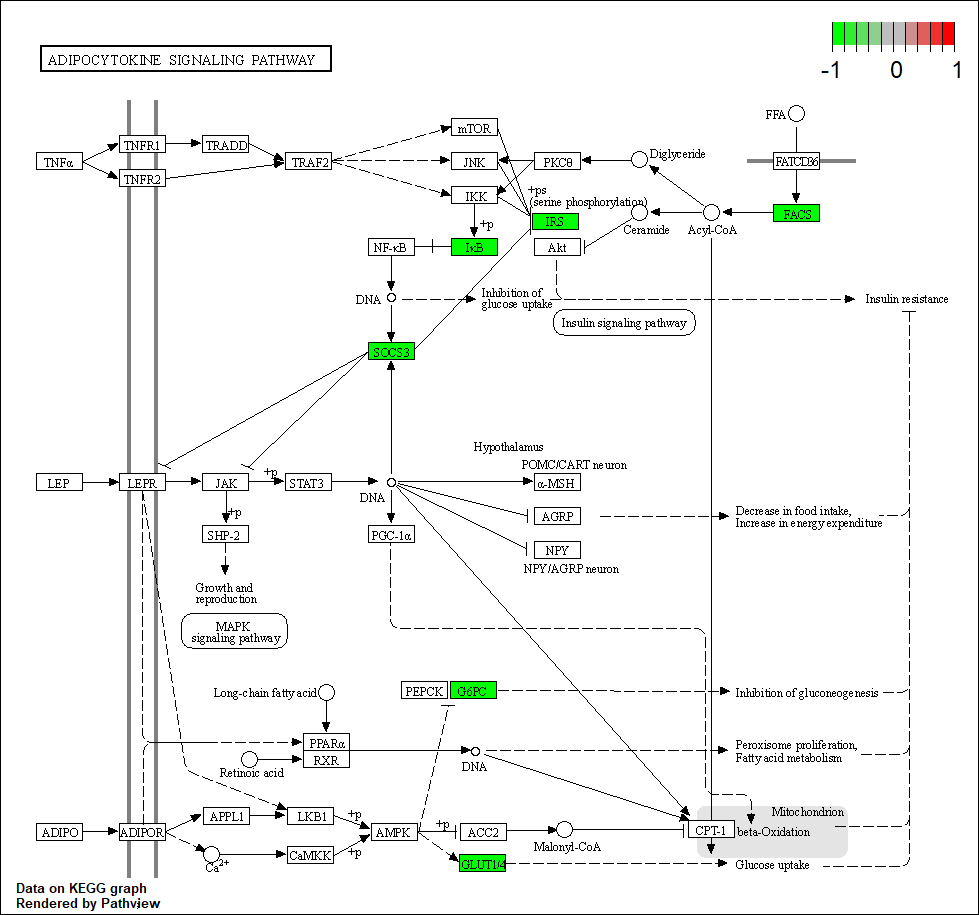

Supplement: Supplementary file 10 [file Image_4.tif]

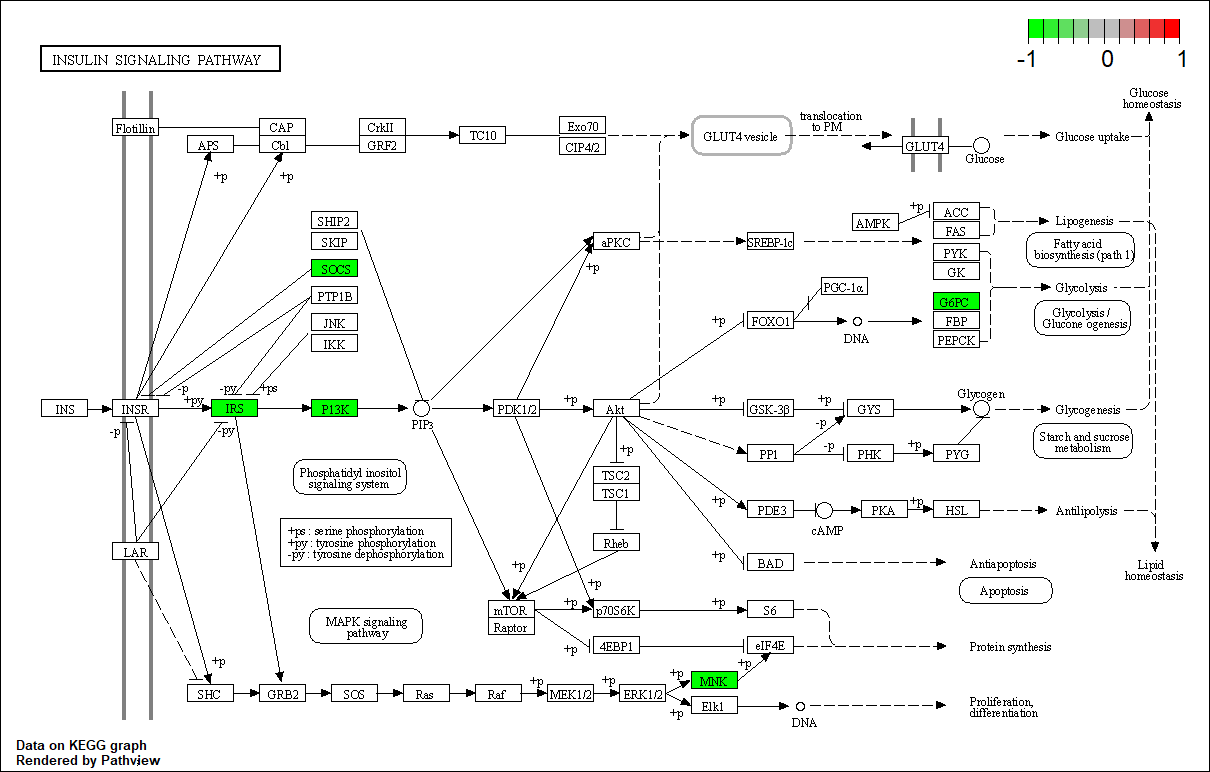

Supplement: Supplementary file 11 [file Image_5.tiff]

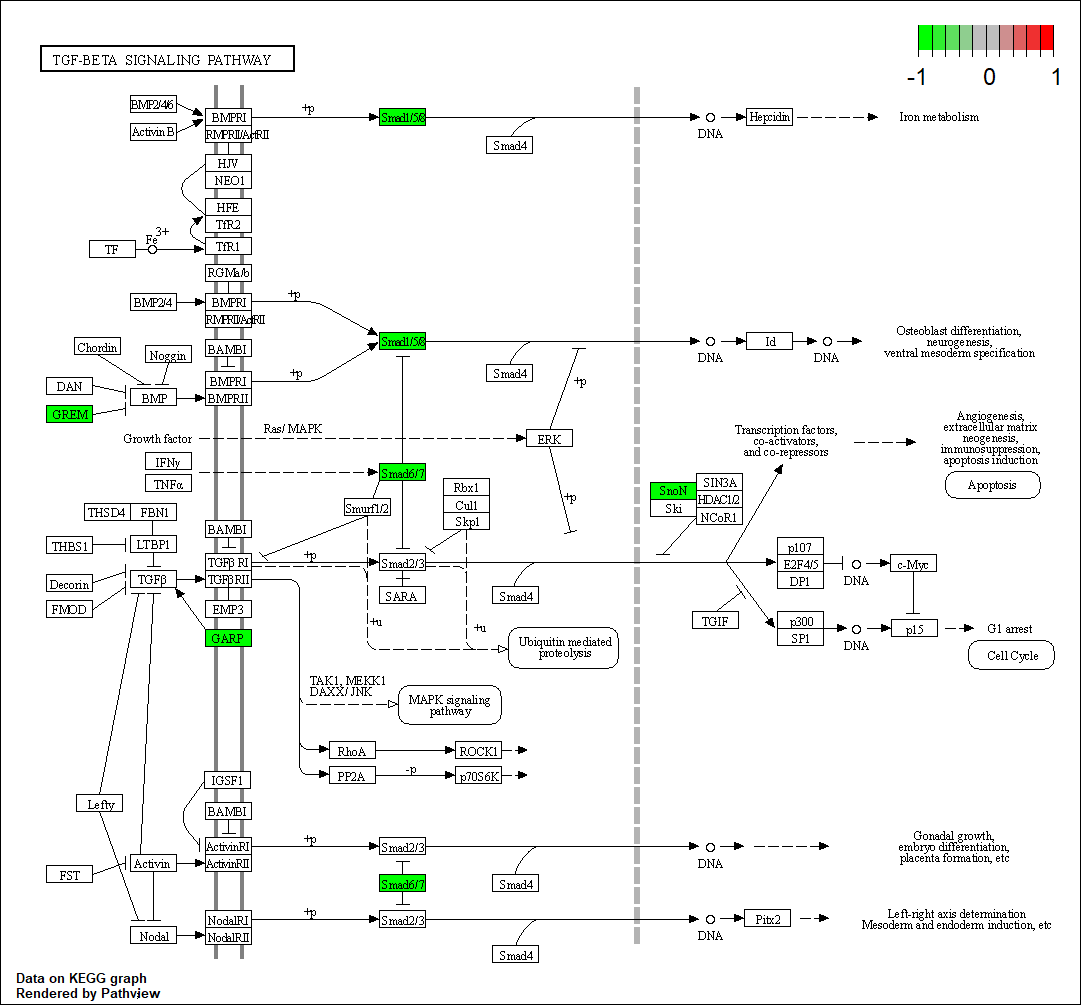

Supplement: Supplementary file 12 [file Image_6.tiff]

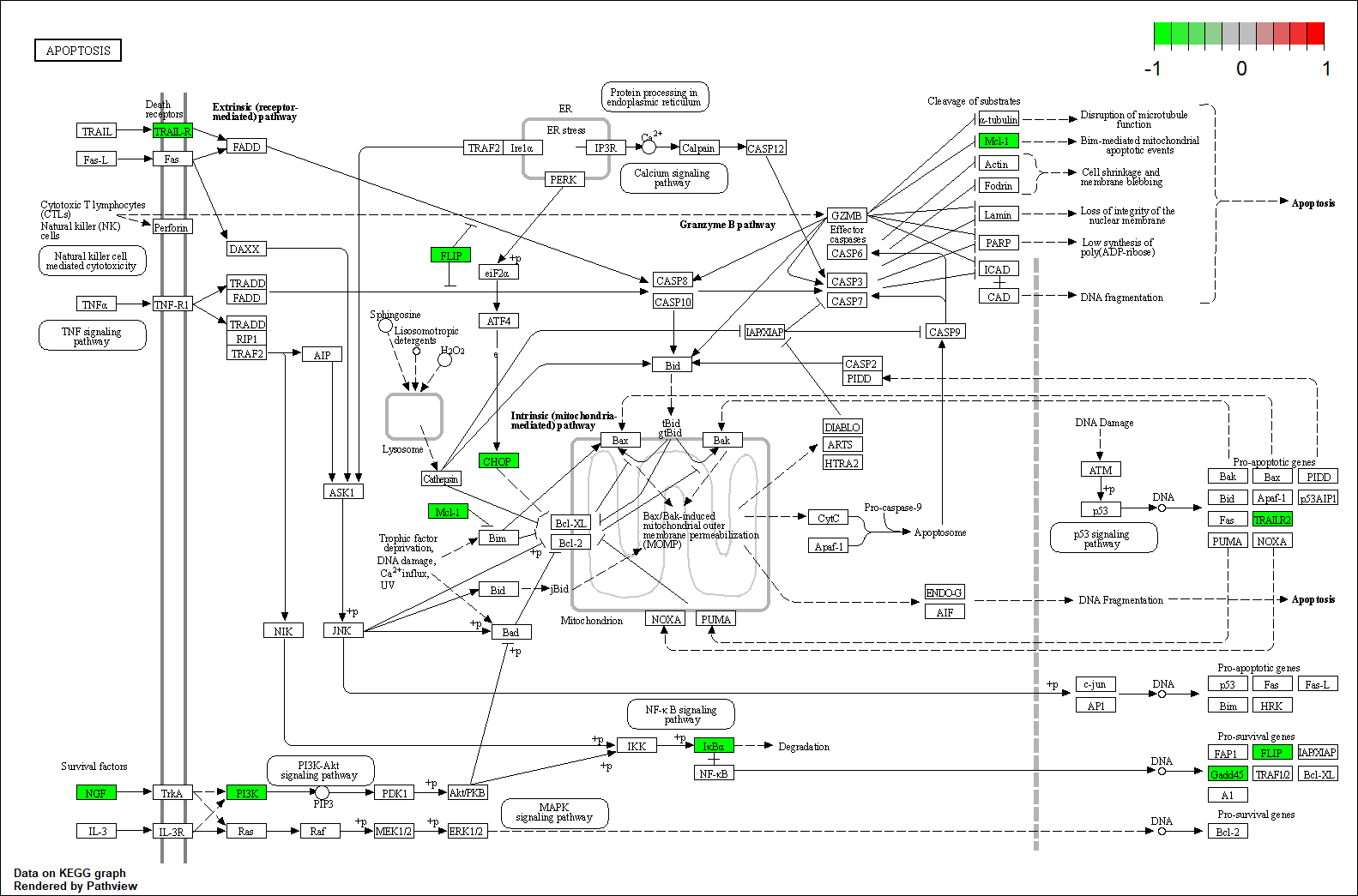

Supplement: Supplementary file 13 [file Image_7.tiff]

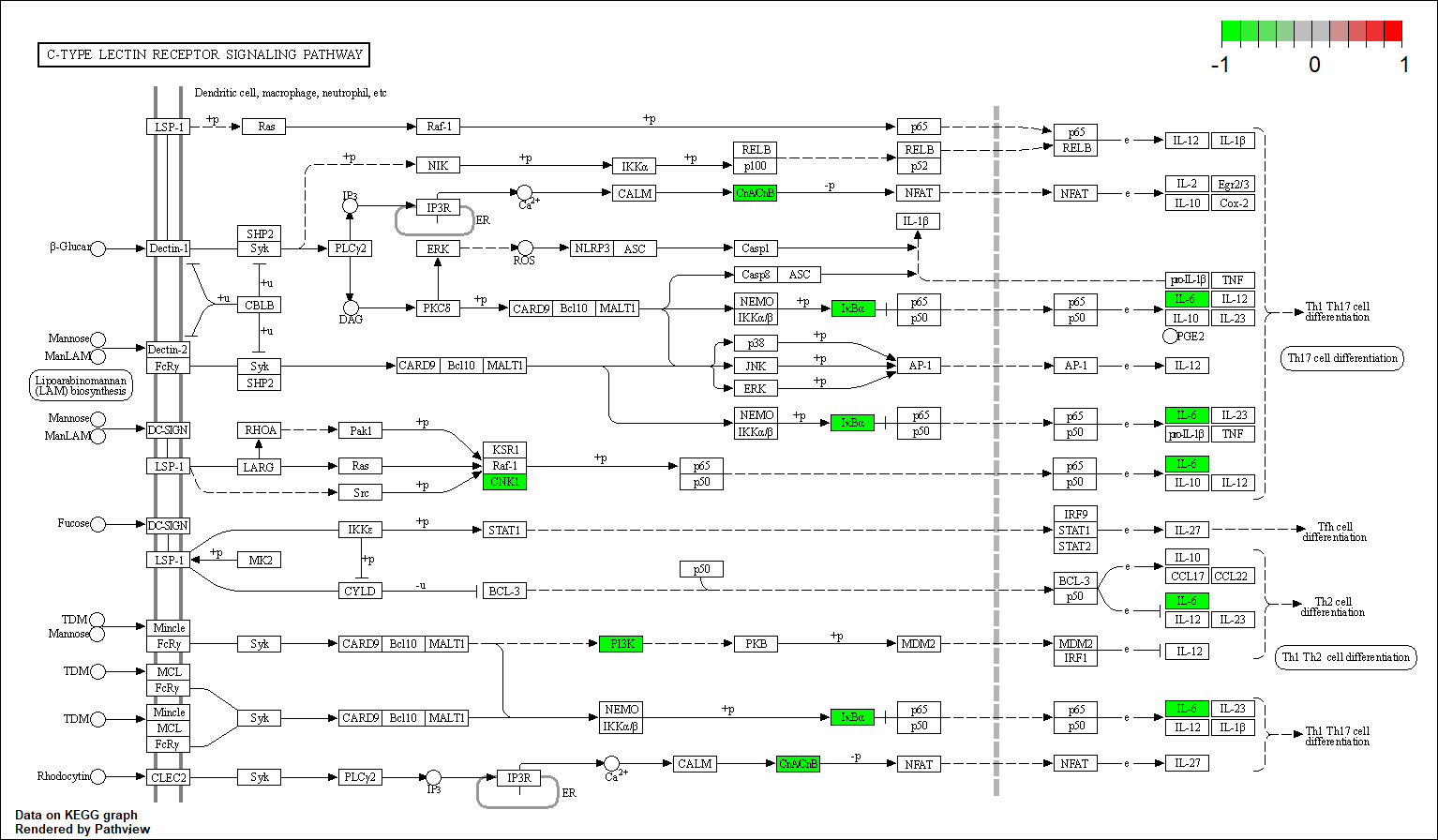

Supplement: Supplementary file 14 [file Image_8.tif]

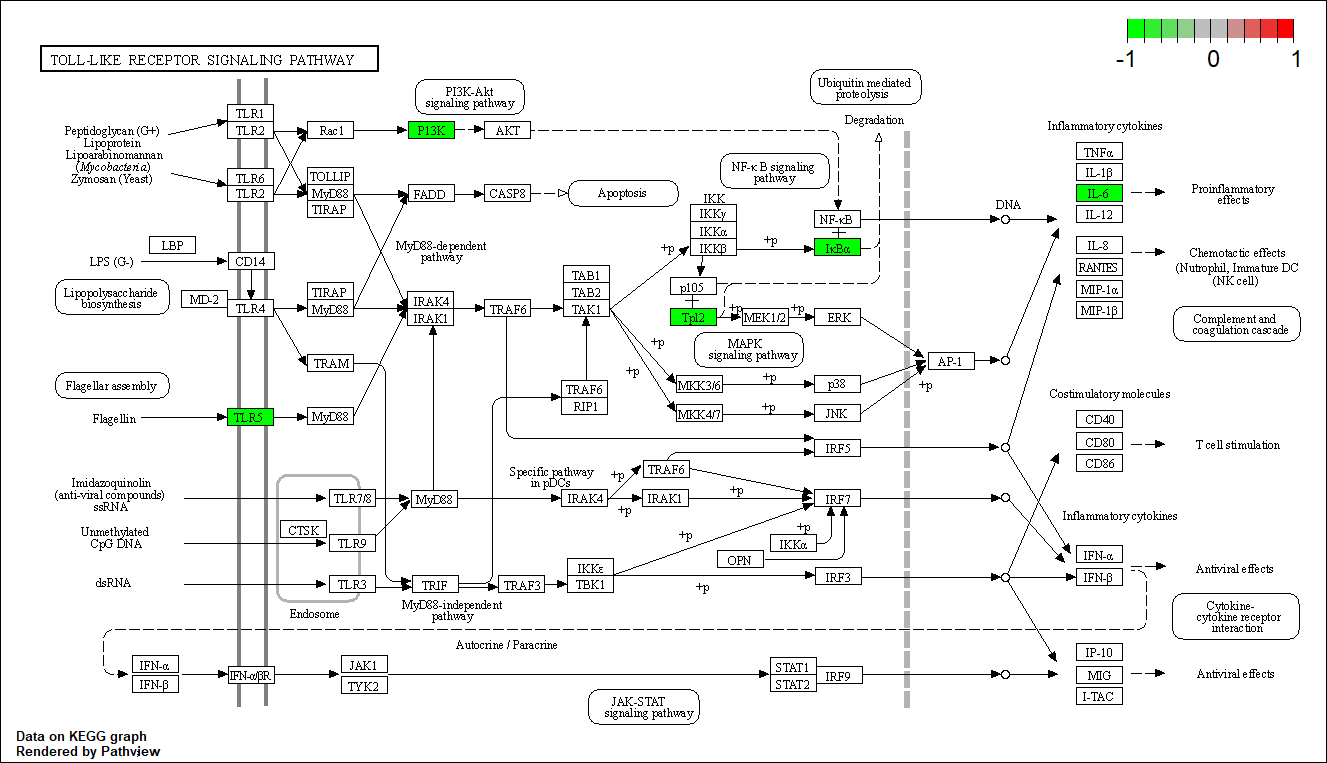

Supplement: Supplementary file 15 [file Image_9.tiff]

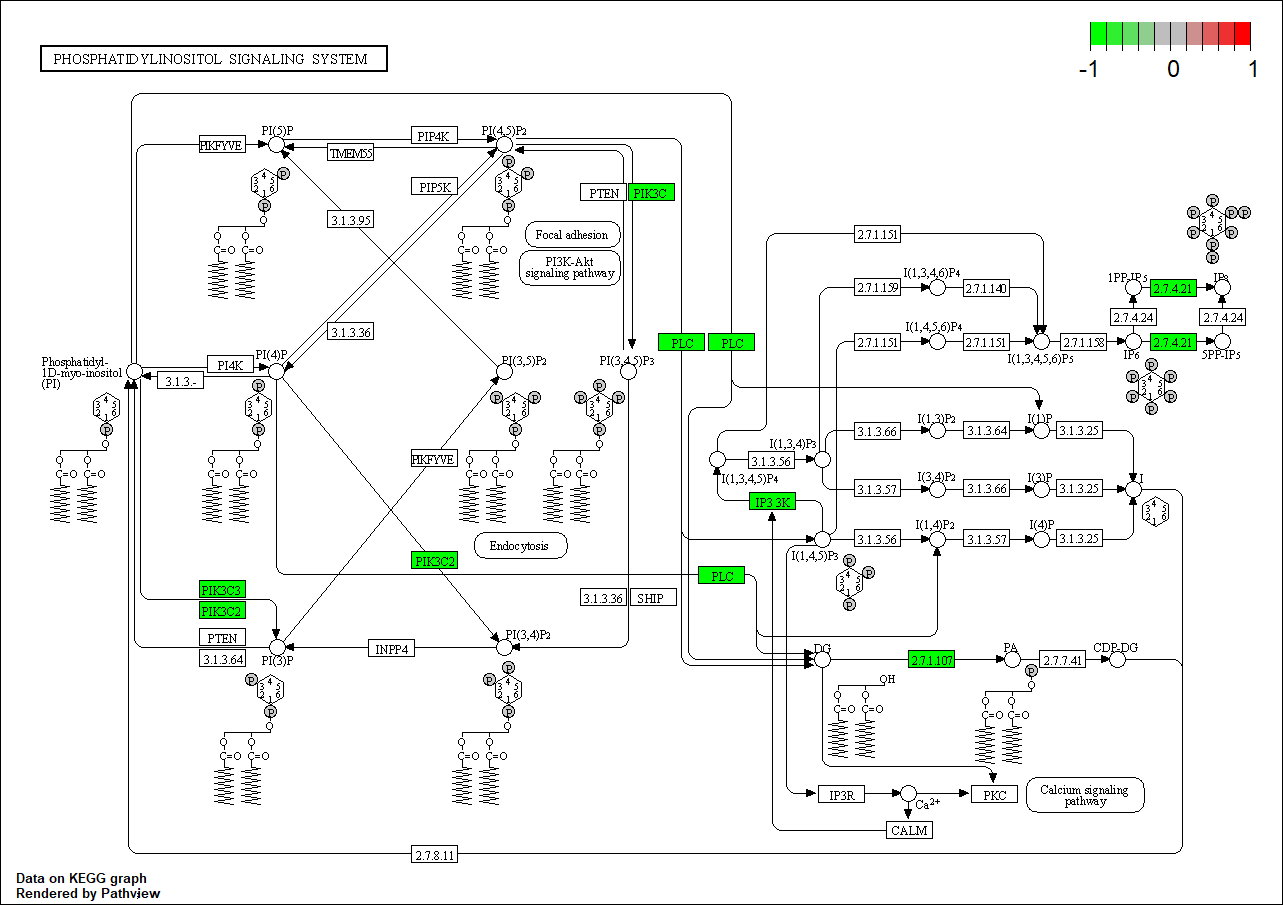

Supplement: Supplementary file 16 [file Image_10.tiff]
